# Supplementary material for: A Panchromatic Cyclometalated Iridium Dye Based on 2-Thienyl-Perimidine
Source: Molecules. 2022 May 17;27(10):3201. doi: 10.3390/molecules27103201 (PMC9143831; doi:10.3390/molecules27103201)

checkCIF/PLATON report

Structure factors have been supplied for datablock(s) bz14b_good

THIS REPORT IS FOR GUIDANCE ONLY. IF USED AS PART OF A REVIEW PROCEDURE FOR PUBLICATION, IT SHOULD NOT REPLACE THE EXPERTISE OF AN EXPERIENCED CRYSTALLOGRAPHIC REFEREE.

No syntax errors found. CIF dictionary Interpreting this report

Datablock: bz14b_good

```
Bond precision:   C-C = 0.0105 A          Wavelength=0.71073

Cell:            a=31.2214(18)     b=12.4801(7)        c=16.8310(9)
                 alpha=90          beta=103.036(2)     gamma=90
Temperature:     100 K
```

	Calculated	Reported
Volume	6389.1(6)	6389.1(6)
Space group	P 21/c	P 1 21/c 1
Hall group	-P 2ybc	-P 2ybc
Moiety formula	C64 H44 Cl2 Ir2 N8 S4 [+ solvent]	C64 H44 Cl2 Ir2 N8 S4
Sum formula	C64 H44 Cl2 Ir2 N8 S4 [+ solvent]	C64 H44 Cl2 Ir2 N8 S4
Mr	1508.66	1508.61
Dx,g cm-3	1.568	1.568
Z	4	4
Mu (mm-1)	4.420	4.420
F000	2944.0	2944.0
F000'	2936.68	
h,k,lmax	45,18,24	45,18,24
Nref	20188	20156
Tmin,Tmax	0.661,0.957	0.586,0.747
Tmin'	0.583	

```
Correction method= # Reported T Limits: Tmin=0.586 Tmax=0.747
AbsCorr = MULTI-SCAN

Data completeness= 0.998           Theta(max)= 30.900
```

R(reflections)= 0.0501(14346)

wR2(reflections)= 0.1145(20156)

S = 1.064 Npar= 725

The following ALERTS were generated. Each ALERT has the format
test-name_ALERT_alert-type_alert-level.
Click on the hyperlinks for more details of the test.

Alert level C

```
PLAT220_ALERT_2_C NonSolvent   Resd 1   C    Ueq(max)/Ueq(min) Range        3.9 Ratio
PLAT342_ALERT_3_C Low Bond Precision on  C-C Bonds ..............     0.01047 Ang.
PLAT431_ALERT_2_C Short Inter HL..A Contact  Cl04      ..S008     .        3.43 Ang.
                                          x,3/2-y,1/2+z  =      4_576 Check
PLAT905_ALERT_3_C Negative K value in the Analysis of Variance ...     -1.122 Report
PLAT911_ALERT_3_C Missing FCF Refl Between Thmin & STh/L=    0.600         10 Report
PLAT977_ALERT_2_C Check Negative Difference Density on H017      .      -0.34 eA-3
PLAT977_ALERT_2_C Check Negative Difference Density on H019      .      -0.37 eA-3
PLAT977_ALERT_2_C Check Negative Difference Density on H01R      .      -0.36 eA-3
PLAT977_ALERT_2_C Check Negative Difference Density on H028      .      -0.34 eA-3
```

Alert level G

```
PLAT083_ALERT_2_G SHELXL Second Parameter in WGHT  Unusually Large      60.23 Why ?
PLAT606_ALERT_4_G Solvent Accessible VOID(S) in Structure ........          ! Info
PLAT720_ALERT_4_G Number of Unusual/Non-Standard Labels ..........        124 Note
PLAT910_ALERT_3_G Missing # of FCF Reflection(s) Below Theta(Min).          2 Note
PLAT912_ALERT_4_G Missing # of FCF Reflections Above STh/L=  0.600         20 Note
PLAT913_ALERT_3_G Missing # of Very Strong Reflections in FCF ....          1 Note
PLAT941_ALERT_3_G Average HKL Measurement Multiplicity ..........         4.0 Low
PLAT967_ALERT_5_G Note: Two-Theta Cutoff Value in Embedded .res ..       61.8 Degree
PLAT978_ALERT_2_G Number C-C Bonds with Positive Residual Density.          0 Info
```

---

0 **ALERT level A** = Most likely a serious problem - resolve or explain
0 **ALERT level B** = A potentially serious problem, consider carefully
9 **ALERT level C** = Check. Ensure it is not caused by an omission or oversight
9 **ALERT level G** = General information/check it is not something unexpected

0 ALERT type 1 CIF construction/syntax error, inconsistent or missing data
8 ALERT type 2 Indicator that the structure model may be wrong or deficient
6 ALERT type 3 Indicator that the structure quality may be low
3 ALERT type 4 Improvement, methodology, query or suggestion
1 ALERT type 5 Informative message, check

It is advisable to attempt to resolve as many as possible of the alerts in all categories. Often the minor alerts point to easily fixed oversights, errors and omissions in your CIF or refinement strategy, so attention to these fine details can be worthwhile. In order to resolve some of the more serious problems it may be necessary to carry out additional measurements or structure refinements. However, the purpose of your study may justify the reported deviations and the more serious of these should normally be commented upon in the discussion or experimental section of a paper or in the "special_details" fields of the CIF. checkCIF was carefully designed to identify outliers and unusual parameters, but every test has its limitations and alerts that are not important in a particular case may appear. Conversely, the absence of alerts does not guarantee there are no aspects of the results needing attention. It is up to the individual to critically assess their own results and, if necessary, seek expert advice.

Publication of your CIF in IUCr journals

A basic structural check has been run on your CIF. These basic checks will be run on all CIFs submitted for publication in IUCr journals (*Acta Crystallographica*, *Journal of Applied Crystallography*, *Journal of Synchrotron Radiation*); however, if you intend to submit to *Acta Crystallographica Section C* or *E* or *IUCrData*, you should make sure that full publication checks are run on the final version of your CIF prior to submission.

Publication of your CIF in other journals

Please refer to the *Notes for Authors* of the relevant journal for any special instructions relating to CIF submission.

PLATON version of 19/02/2022; check.def file version of 19/02/2022

Datablock bz14b_good - ellipsoid plot

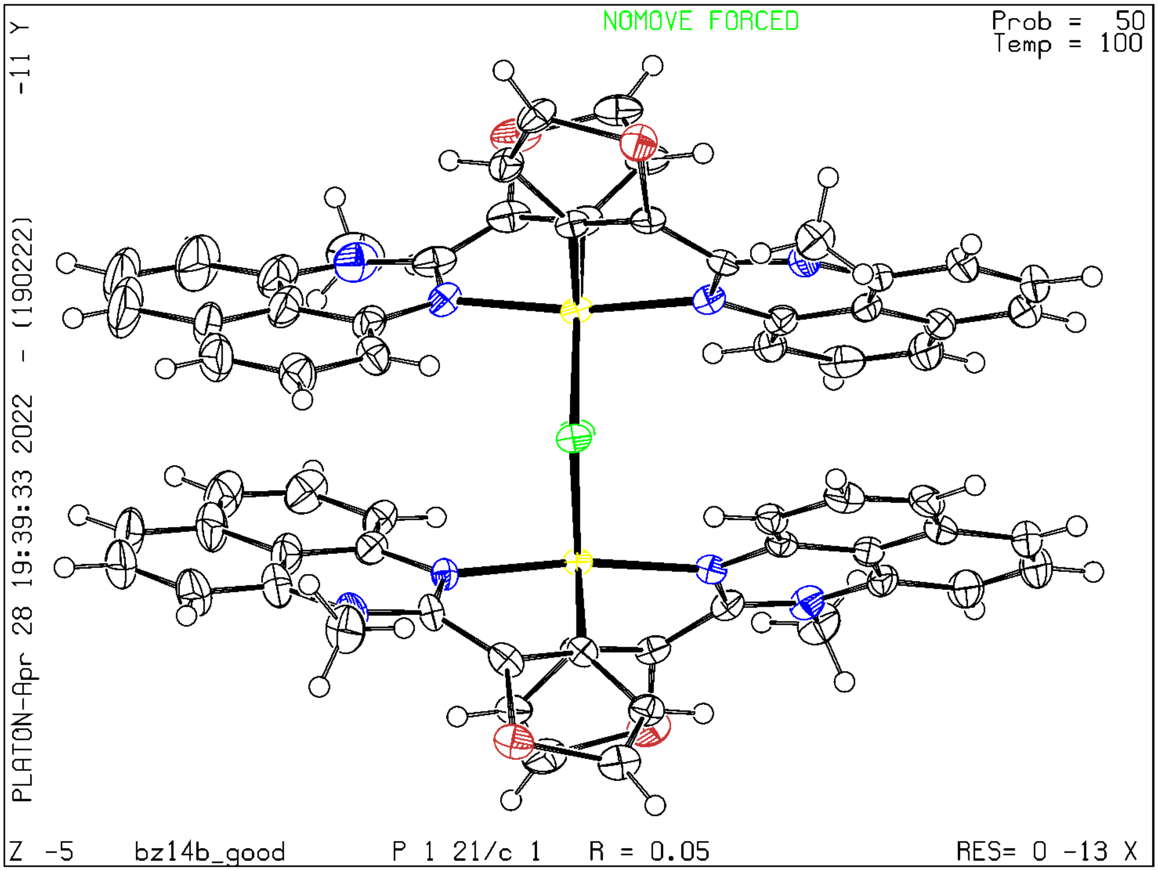

Supplement: Supplementary file 1 [file molecules-27-03201-s001.zip › checkcif 1.pdf]
