# Supplementary material for: A Panchromatic Cyclometalated Iridium Dye Based on 2-Thienyl-Perimidine
Source: Molecules. 2022 May 17;27(10):3201. doi: 10.3390/molecules27103201 (PMC9143831; doi:10.3390/molecules27103201)

checkCIF/PLATON report

Structure factors have been supplied for datablock(s) bz15neo_1

THIS REPORT IS FOR GUIDANCE ONLY. IF USED AS PART OF A REVIEW PROCEDURE FOR PUBLICATION, IT SHOULD NOT REPLACE THE EXPERTISE OF AN EXPERIENCED CRYSTALLOGRAPHIC REFEREE.

No syntax errors found. CIF dictionary Interpreting this report

Datablock: bz15neo_1

```
Bond precision:   C-C = 0.0032 A           Wavelength=0.71073

Cell:           a=11.3183(5)       b=13.0884(7)       c=20.3399(9)
                alpha=106.294(2)   beta=103.709(1)    gamma=90.068(2)
Temperature:    100 K
```

	Calculated	Reported
Volume	2802.3(2)	2802.3(2)
Space group	P -1	P -1
Hall group	-P 1	-P 1
Moiety formula	C44 H30 Ir N6 O4 S2, F6 P, 3(C H2 Cl2), 3(C H4 O)	C44 H30 Ir N6 O4 S2, F6 P, 3(C H2 Cl2), 3(C H4 O)
Sum formula	C50 H48 Cl6 F6 Ir N6 O7 P S2	C50 H48 Cl6 F6 Ir N6 O7 P S2
Mr	1458.95	1458.93
Dx,g cm-3	1.729	1.729
Z	2	2
Mu (mm-1)	2.848	2.848
F000	1452.0	1452.0
F000’	1452.22	
h,k,lmax	17,20,31	17,20,31
Nref	22421	21801
Tmin,Tmax	0.565,0.652	0.603,0.747
Tmin’	0.500	

```
Correction method= # Reported T Limits: Tmin=0.603 Tmax=0.747
AbsCorr = MULTI-SCAN

Data completeness= 0.972            Theta(max)= 33.747
```

R(reflections)= 0.0300(19979)

wR2(reflections)= 0.0758(21801)

S = 1.041 Npar= 725

The following ALERTS were generated. Each ALERT has the format
test-name_ALERT_alert-type_alert-level.
Click on the hyperlinks for more details of the test.

Alert level C

```
PLAT243_ALERT_4_C High   'Solvent' Ueq as Compared to Neighbors of       C027 Check
PLAT244_ALERT_4_C Low    'Solvent' Ueq as Compared to Neighbors of       P004 Check
PLAT250_ALERT_2_C Large U3/U1 Ratio for Average U(i,j) Tensor ....        2.2 Note
PLAT417_ALERT_2_C Short Inter D-H..H-D       H00K      ..H00L     .      2.14 Ang.
                                                  x,y,z  =      1_555 Check
PLAT911_ALERT_3_C Missing FCF Refl Between Thmin & STh/L=    0.600          3 Report
PLAT971_ALERT_2_C Check Calcd Resid. Dens.  0.68Ang From Ir01            2.23 eA-3
PLAT972_ALERT_2_C Check Calcd Resid. Dens.  0.59Ang From Cl2            -1.85 eA-3
PLAT972_ALERT_2_C Check Calcd Resid. Dens.  0.45Ang From Cl2            -1.66 eA-3
PLAT972_ALERT_2_C Check Calcd Resid. Dens.  0.39Ang From Cl2            -1.53 eA-3
PLAT972_ALERT_2_C Check Calcd Resid. Dens.  0.50Ang From ClB            -1.53 eA-3
```

Alert level G

```
PLAT007_ALERT_5_G Number of Unrefined Donor-H Atoms ..............          5 Report
PLAT171_ALERT_4_G The CIF-Embedded .res File Contains EADP Records          1 Report
PLAT300_ALERT_4_G Atom Site Occupancy of ClB        Constrained at        0.5 Check
PLAT300_ALERT_4_G Atom Site Occupancy of ClA        Constrained at        0.5 Check
PLAT300_ALERT_4_G Atom Site Occupancy of H02A       Constrained at        0.5 Check
PLAT300_ALERT_4_G Atom Site Occupancy of H02B       Constrained at        0.5 Check
PLAT300_ALERT_4_G Atom Site Occupancy of H02C       Constrained at        0.5 Check
PLAT300_ALERT_4_G Atom Site Occupancy of H02D       Constrained at        0.5 Check
PLAT302_ALERT_4_G Anion/Solvent/Minor-Residue Disorder (Resd  3  )        33% Note
PLAT432_ALERT_2_G Short Inter X...Y Contact  O00K      ..C01L     .      2.92 Ang.
                                             -x,1-y,1-z  =      2_566 Check
PLAT720_ALERT_4_G Number of Unusual/Non-Standard Labels ..........        125 Note
PLAT910_ALERT_3_G Missing # of FCF Reflection(s) Below Theta(Min).          4 Note
PLAT912_ALERT_4_G Missing # of FCF Reflections Above STh/L=  0.600        613 Note
PLAT933_ALERT_2_G Number of HKL-OMIT Records in Embedded .res File          4 Note
PLAT978_ALERT_2_G Number C-C Bonds with Positive Residual Density.          3 Info
```

---

0 **ALERT level A** = Most likely a serious problem - resolve or explain
0 **ALERT level B** = A potentially serious problem, consider carefully
10 **ALERT level C** = Check. Ensure it is not caused by an omission or oversight
15 **ALERT level G** = General information/check it is not something unexpected

0 ALERT type 1 CIF construction/syntax error, inconsistent or missing data
10 ALERT type 2 Indicator that the structure model may be wrong or deficient
2 ALERT type 3 Indicator that the structure quality may be low
12 ALERT type 4 Improvement, methodology, query or suggestion
1 ALERT type 5 Informative message, check

It is advisable to attempt to resolve as many as possible of the alerts in all categories. Often the minor alerts point to easily fixed oversights, errors and omissions in your CIF or refinement strategy, so attention to these fine details can be worthwhile. In order to resolve some of the more serious problems it may be necessary to carry out additional measurements or structure refinements. However, the purpose of your study may justify the reported deviations and the more serious of these should normally be commented upon in the discussion or experimental section of a paper or in the "special_details" fields of the CIF. checkCIF was carefully designed to identify outliers and unusual parameters, but every test has its limitations and alerts that are not important in a particular case may appear. Conversely, the absence of alerts does not guarantee there are no aspects of the results needing attention. It is up to the individual to critically assess their own results and, if necessary, seek expert advice.

Publication of your CIF in IUCr journals

A basic structural check has been run on your CIF. These basic checks will be run on all CIFs submitted for publication in IUCr journals (*Acta Crystallographica*, *Journal of Applied Crystallography*, *Journal of Synchrotron Radiation*); however, if you intend to submit to *Acta Crystallographica Section C* or *E* or *IUCrData*, you should make sure that full publication checks are run on the final version of your CIF prior to submission.

Publication of your CIF in other journals

Please refer to the *Notes for Authors* of the relevant journal for any special instructions relating to CIF submission.

PLATON version of 19/02/2022; check.def file version of 19/02/2022

Datablock bz15neo_1 - ellipsoid plot

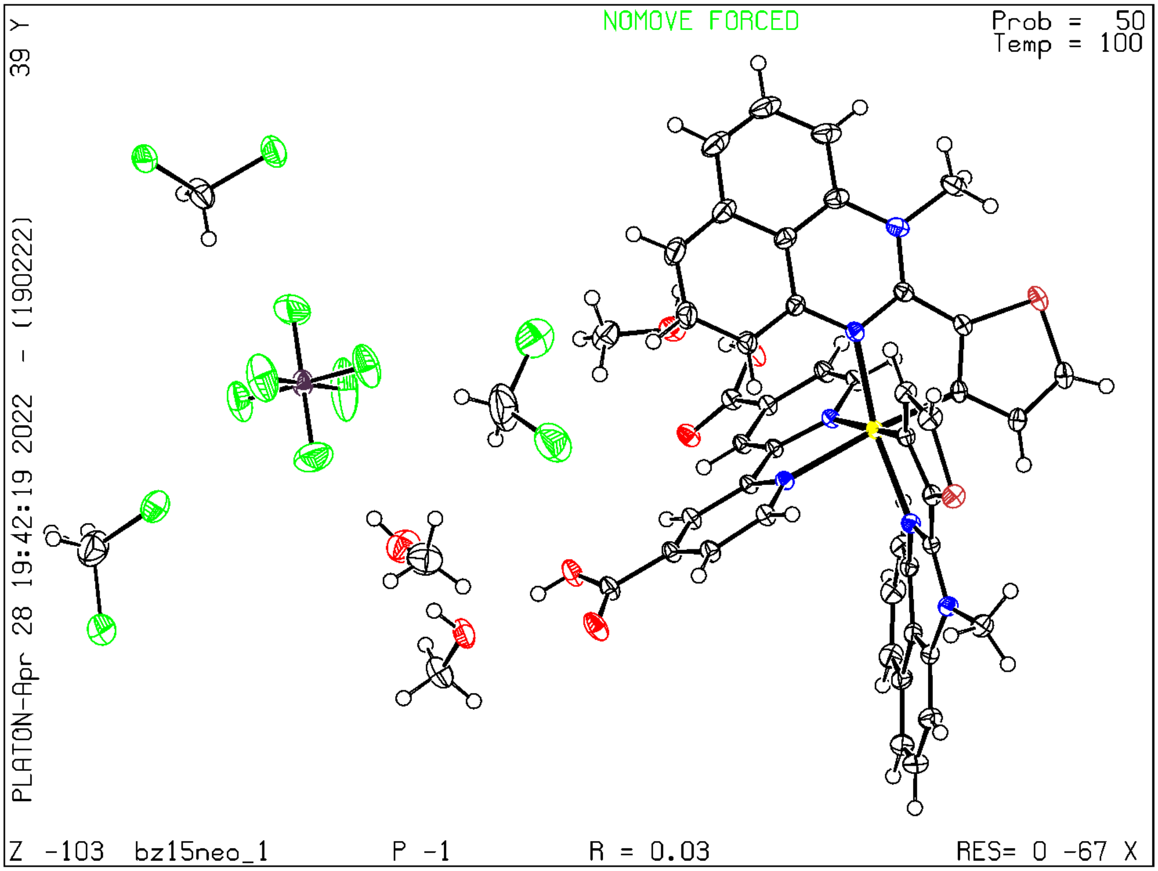

Supplement: Supplementary file 1 [file molecules-27-03201-s001.zip › checkcif 2.pdf]
