# Supplementary material for: A Panchromatic Cyclometalated Iridium Dye Based on 2-Thienyl-Perimidine
Source: Molecules. 2022 May 17;27(10):3201. doi: 10.3390/molecules27103201 (PMC9143831; doi:10.3390/molecules27103201)

checkCIF/PLATON report

Structure factors have been supplied for datablock(s) mk12b

THIS REPORT IS FOR GUIDANCE ONLY. IF USED AS PART OF A REVIEW PROCEDURE FOR PUBLICATION, IT SHOULD NOT REPLACE THE EXPERTISE OF AN EXPERIENCED CRYSTALLOGRAPHIC REFEREE.

No syntax errors found. CIF dictionary Interpreting this report

Datablock: mk12b

```
Bond precision:   C-C = 0.0017 A          Wavelength=0.71073

Cell:              a=7.7813(4)      b=11.5947(5)       c=13.6577(6)
                   alpha=90         beta=93.067(2)     gamma=90
Temperature:       100 K

                 Calculated                   Reported
Volume           1230.46(10)                  1230.46(10)
Space group      P 21/c                       P 1 21/c 1
Hall group       -P 2ybc                      -P 2ybc
Moiety formula   C16 H12 N2 S                 C16 H12 N2 S
Sum formula      C16 H12 N2 S                 C16 H12 N2 S
Mr               264.34                       264.34
Dx,g cm-3        1.427                        1.427
Z                4                            4
Mu (mm-1)        0.248                        0.248
F000             552.0                        552.0
F000'            552.66
h,k,lmax         10,16,18                     10,16,18
Nref             3426                         3416
Tmin,Tmax        0.942,0.961                  0.706,0.746
Tmin'            0.940

Correction method= # Reported T Limits: Tmin=0.706 Tmax=0.746
AbsCorr = MULTI-SCAN

Data completeness= 0.997          Theta(max)= 29.498

                                                     wR2(reflections)=
R(reflections)= 0.0394( 2768)                        0.1023( 3416)

S = 1.036                  Npar= 191
```

The following ALERTS were generated. Each ALERT has the format
test-name_ALERT_alert-type_alert-level.
Click on the hyperlinks for more details of the test.

Alert level C

```
PLAT906_ALERT_3_C Large K Value in the Analysis of Variance ......      2.032 Check
PLAT911_ALERT_3_C Missing FCF Refl Between Thmin & STh/L=    0.600         10 Report
PLAT913_ALERT_3_C Missing # of Very Strong Reflections in FCF ....          8 Note
```

Alert level G

```
PLAT002_ALERT_2_G Number of Distance or Angle Restraints on AtSite          3 Note
PLAT176_ALERT_4_G The CIF-Embedded .res File Contains SADI Records          1 Report
PLAT230_ALERT_2_G Hirshfeld Test Diff for    S1       --C3       .       13.4 s.u.
PLAT230_ALERT_2_G Hirshfeld Test Diff for    S2       --C2       .        9.6 s.u.
PLAT230_ALERT_2_G Hirshfeld Test Diff for    C1       --C5       .        9.5 s.u.
PLAT300_ALERT_4_G Atom Site Occupancy of S1         Constrained at       0.55 Check
PLAT300_ALERT_4_G Atom Site Occupancy of S2         Constrained at       0.45 Check
PLAT300_ALERT_4_G Atom Site Occupancy of C1         Constrained at       0.55 Check
PLAT300_ALERT_4_G Atom Site Occupancy of C4         Constrained at       0.45 Check
PLAT300_ALERT_4_G Atom Site Occupancy of H1         Constrained at       0.55 Check
PLAT300_ALERT_4_G Atom Site Occupancy of H2A        Constrained at       0.55 Check
PLAT300_ALERT_4_G Atom Site Occupancy of H3A        Constrained at       0.55 Check
PLAT300_ALERT_4_G Atom Site Occupancy of H2B        Constrained at       0.45 Check
PLAT300_ALERT_4_G Atom Site Occupancy of H3B        Constrained at       0.45 Check
PLAT300_ALERT_4_G Atom Site Occupancy of H4         Constrained at       0.45 Check
PLAT301_ALERT_3_G Main Residue  Disorder .............(Resd  1  )         11% Note
PLAT860_ALERT_3_G Number of Least-Squares Restraints ............           1 Note
PLAT883_ALERT_1_G No Info/Value for _atom_sites_solution_primary .     Please Do !
PLAT912_ALERT_4_G Missing # of FCF Reflections Above STh/L=  0.600          1 Note
PLAT941_ALERT_3_G Average HKL Measurement Multiplicity ..........         4.1 Low
PLAT967_ALERT_5_G Note: Two-Theta Cutoff Value in Embedded .res ..       59.0 Degree
PLAT978_ALERT_2_G Number C-C Bonds with Positive Residual Density.         16 Info
```

0 **ALERT level A** = Most likely a serious problem - resolve or explain
0 **ALERT level B** = A potentially serious problem, consider carefully
3 **ALERT level C** = Check. Ensure it is not caused by an omission or oversight
22 **ALERT level G** = General information/check it is not something unexpected

1 ALERT type 1 CIF construction/syntax error, inconsistent or missing data
5 ALERT type 2 Indicator that the structure model may be wrong or deficient
6 ALERT type 3 Indicator that the structure quality may be low
12 ALERT type 4 Improvement, methodology, query or suggestion
1 ALERT type 5 Informative message, check

It is advisable to attempt to resolve as many as possible of the alerts in all categories. Often the minor alerts point to easily fixed oversights, errors and omissions in your CIF or refinement strategy, so attention to these fine details can be worthwhile. In order to resolve some of the more serious problems it may be necessary to carry out additional measurements or structure refinements. However, the purpose of your study may justify the reported deviations and the more serious of these should normally be commented upon in the discussion or experimental section of a paper or in the "special_details" fields of the CIF. checkCIF was carefully designed to identify outliers and unusual parameters, but every test has its limitations and alerts that are not important in a particular case may appear. Conversely, the absence of alerts does not guarantee there are no aspects of the results needing attention. It is up to the individual to critically assess their own results and, if necessary, seek expert advice.

Publication of your CIF in IUCr journals

A basic structural check has been run on your CIF. These basic checks will be run on all CIFs submitted for publication in IUCr journals (*Acta Crystallographica*, *Journal of Applied Crystallography*, *Journal of Synchrotron Radiation*); however, if you intend to submit to *Acta Crystallographica Section C* or *E* or *IUCrData*, you should make sure that full publication checks are run on the final version of your CIF prior to submission.

Publication of your CIF in other journals

Please refer to the *Notes for Authors* of the relevant journal for any special instructions relating to CIF submission.

PLATON version of 19/02/2022; check.def file version of 19/02/2022

Datablock mk12b - ellipsoid plot

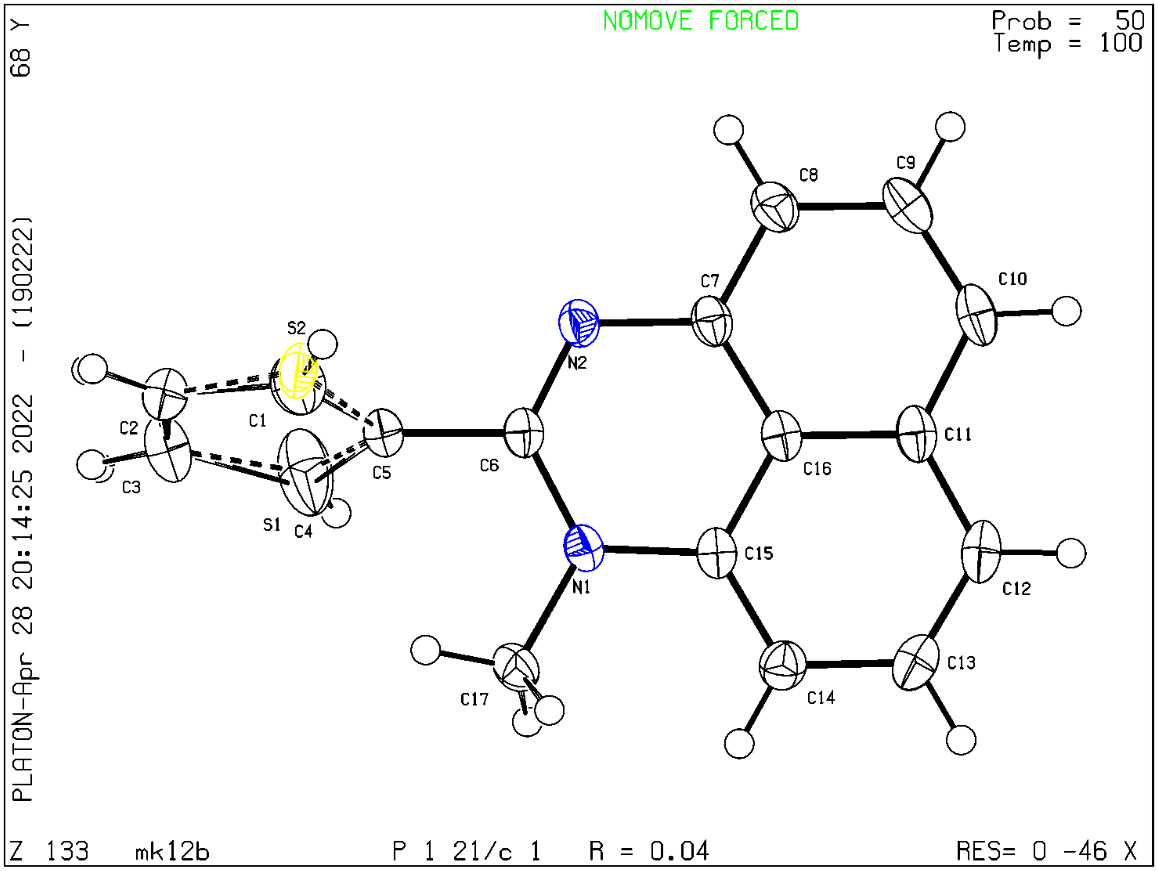

Supplement: Supplementary file 1 [file molecules-27-03201-s001.zip › checkcif L2.pdf]
