# Supplementary material for: A Panchromatic Cyclometalated Iridium Dye Based on 2-Thienyl-Perimidine
Source: Molecules. 2022 May 17;27(10):3201. doi: 10.3390/molecules27103201 (PMC9143831; doi:10.3390/molecules27103201)
Supplement: Supplementary file 1 [file molecules-27-03201-s001.zip › molecules-1727601- Supplementary data-revised.pdf]

Supplementary material

A panchromic cyclometalated iridium dye based on 2-thienyl-perimidine

Paulina Kalle, Marina A. Kiseleva, Sergei V. Tatarin, Daniil E. Smirnov, Alexander Y. Zakharov, Viktor V. Emets, Andrei V. Churakov, and Stanislav I. Bezzubov

Part 1. NMR spectroscopy and high-resolution mass-spectrometry data: Figures S1–S12;

Part 2. X-ray crystallography: Tables S1–S4, Figures S13–S20;

Part 3. Electrochemical and photovoltaic data: Figures S21–S27;

Part 4. Computational results: Table S5, Figures S28–S30.

1. *NMR and high-resolution mass-spectra*

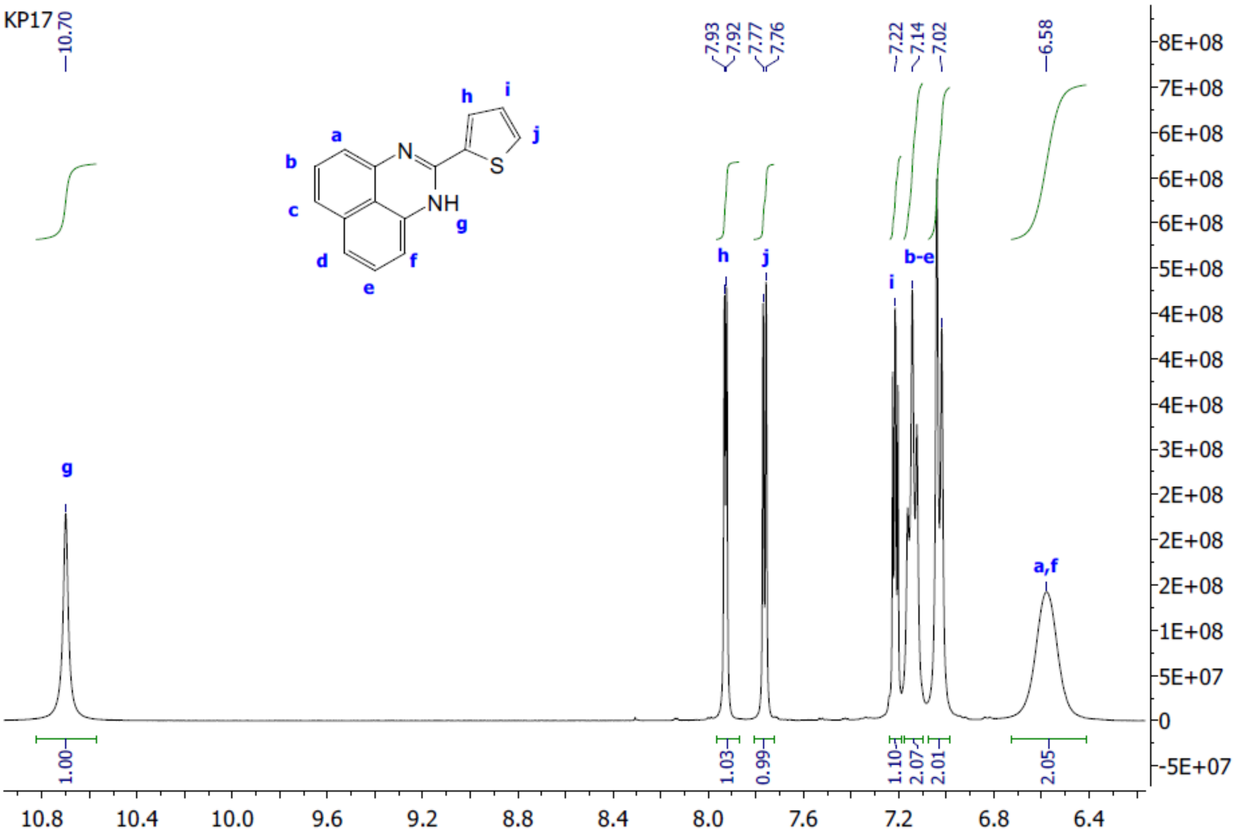

Figure S1. The aromatic part of ^{1}H NMR spectrum of **L1** ($CDCl_3$, 400 MHz, 25°C).

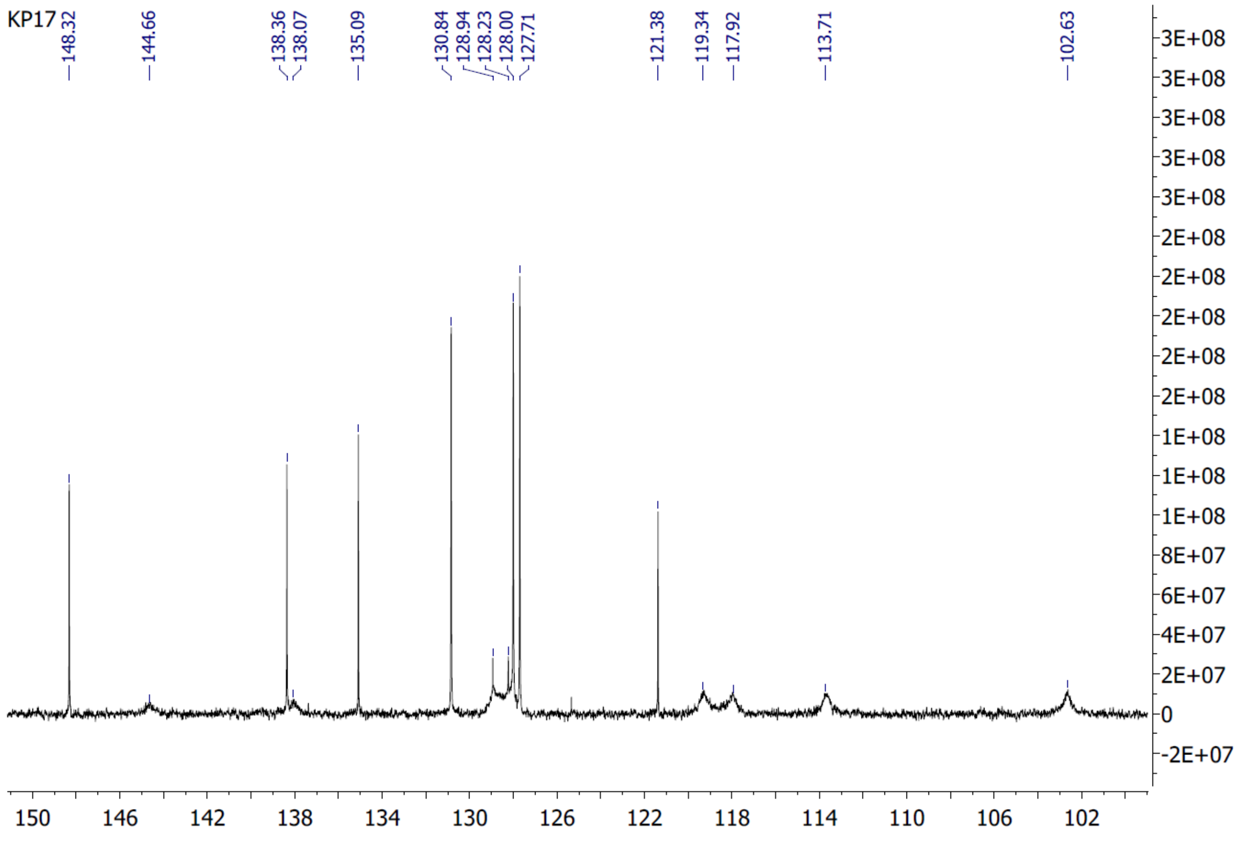

Figure S2. $^{13}C\{^{1}H\}$ NMR spectrum of **L1** ($CDCl_3$, 101 MHz, 25°C).

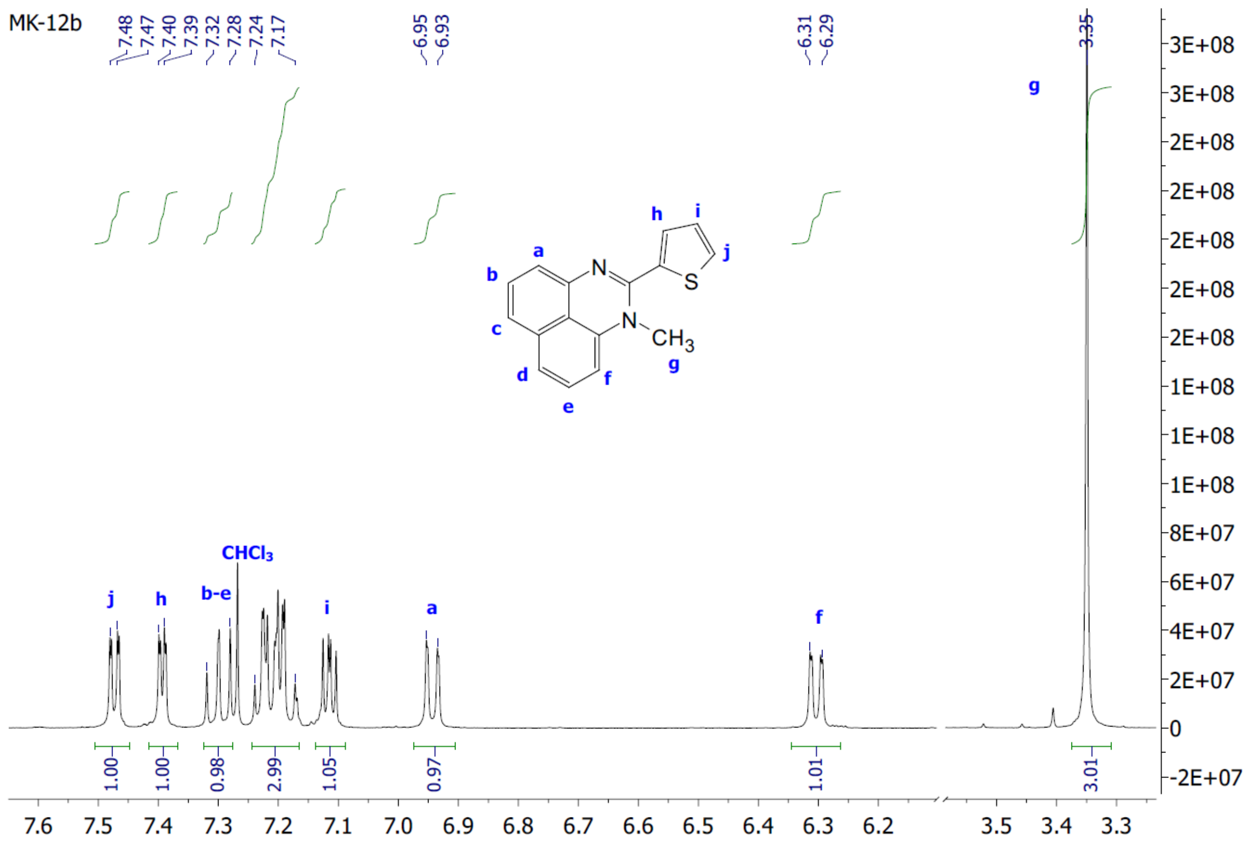

Figure S3. ^{1}H NMR spectrum of **L2** ($CDCl_3$, 400 MHz, 25°C).

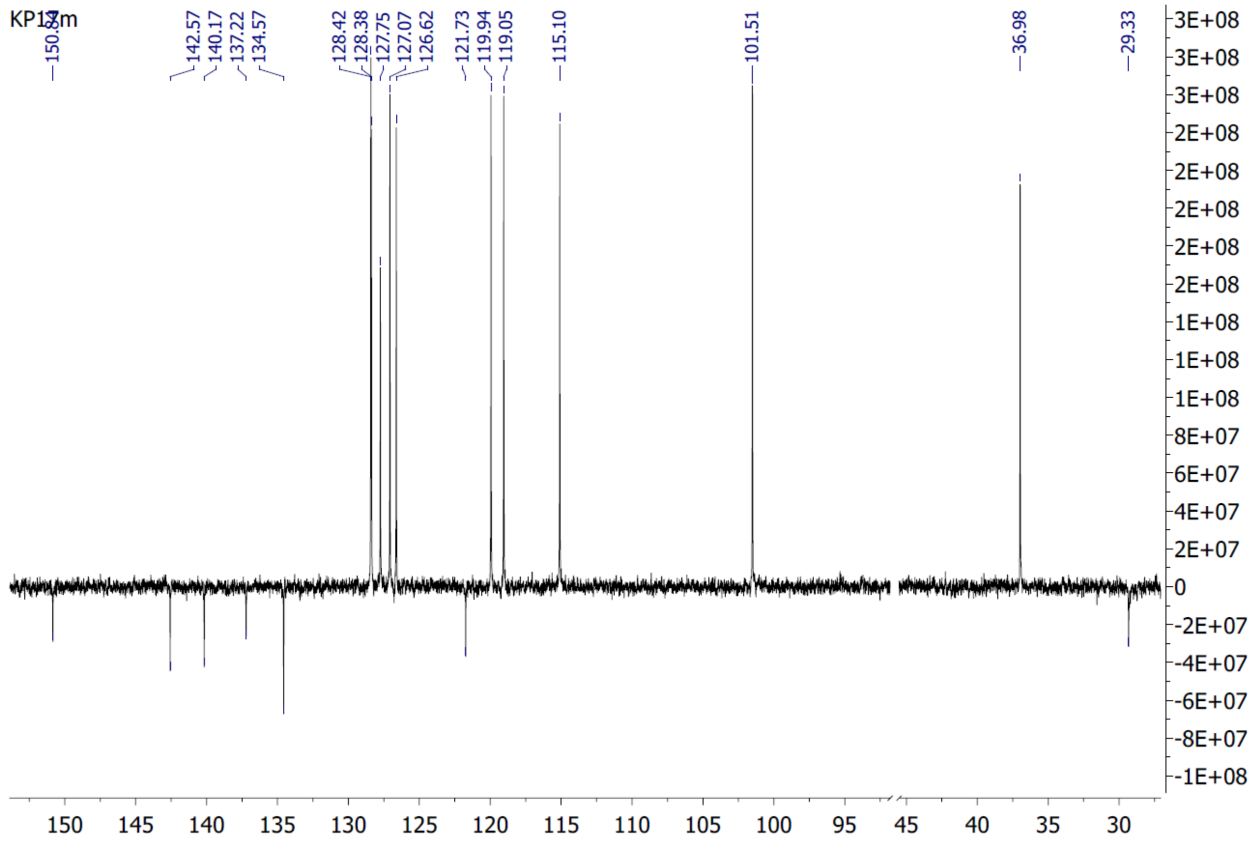

Figure S4. ^{13}C{^{1}H} NMR spectrum of **L2** ($CDCl_3$, 101 MHz, 25°C).

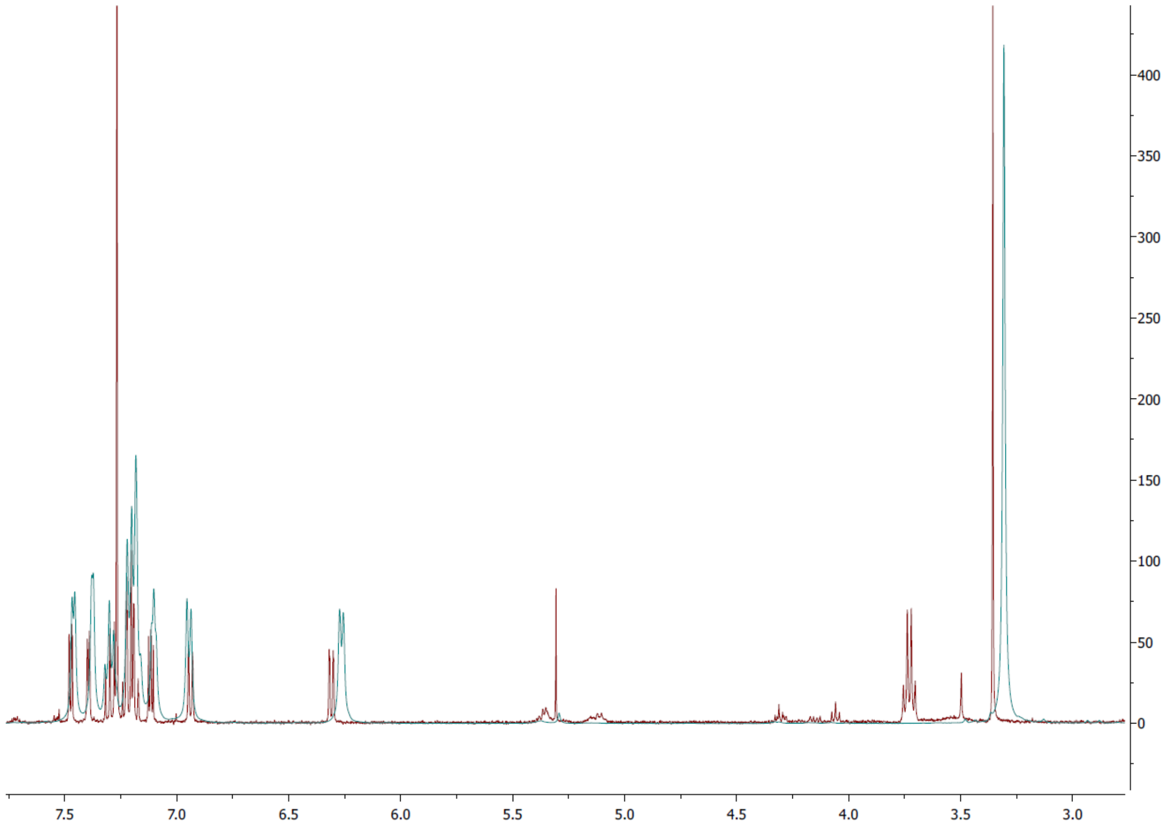

Figure S5. ¹H NMR spectrum of the reaction mixture (*red*) and the ligand **L2** (*blue*) ($CDCl_3$, 400 MHz, 25°C).

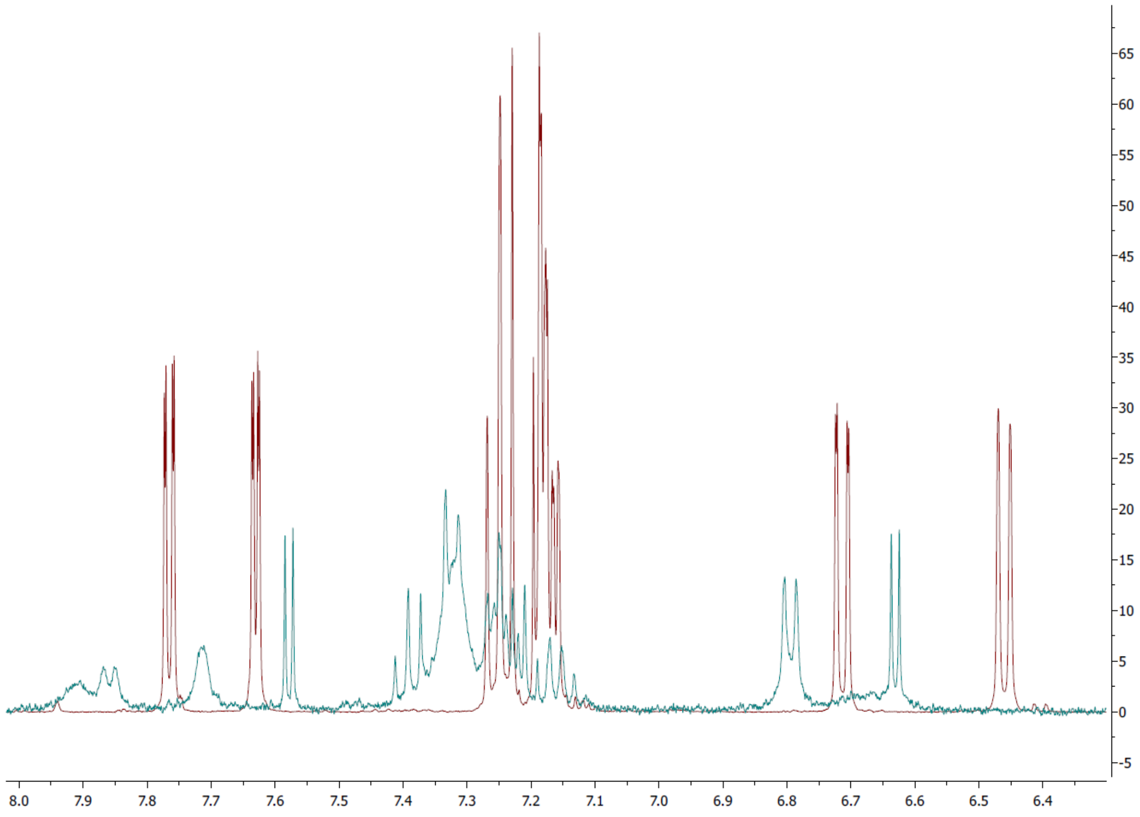

Figure S6. The aromatic part of ¹H NMR spectrum of **1** (*blue*) and **L2** (*red*) (DMSO-d_6, 400 MHz, 25°C).

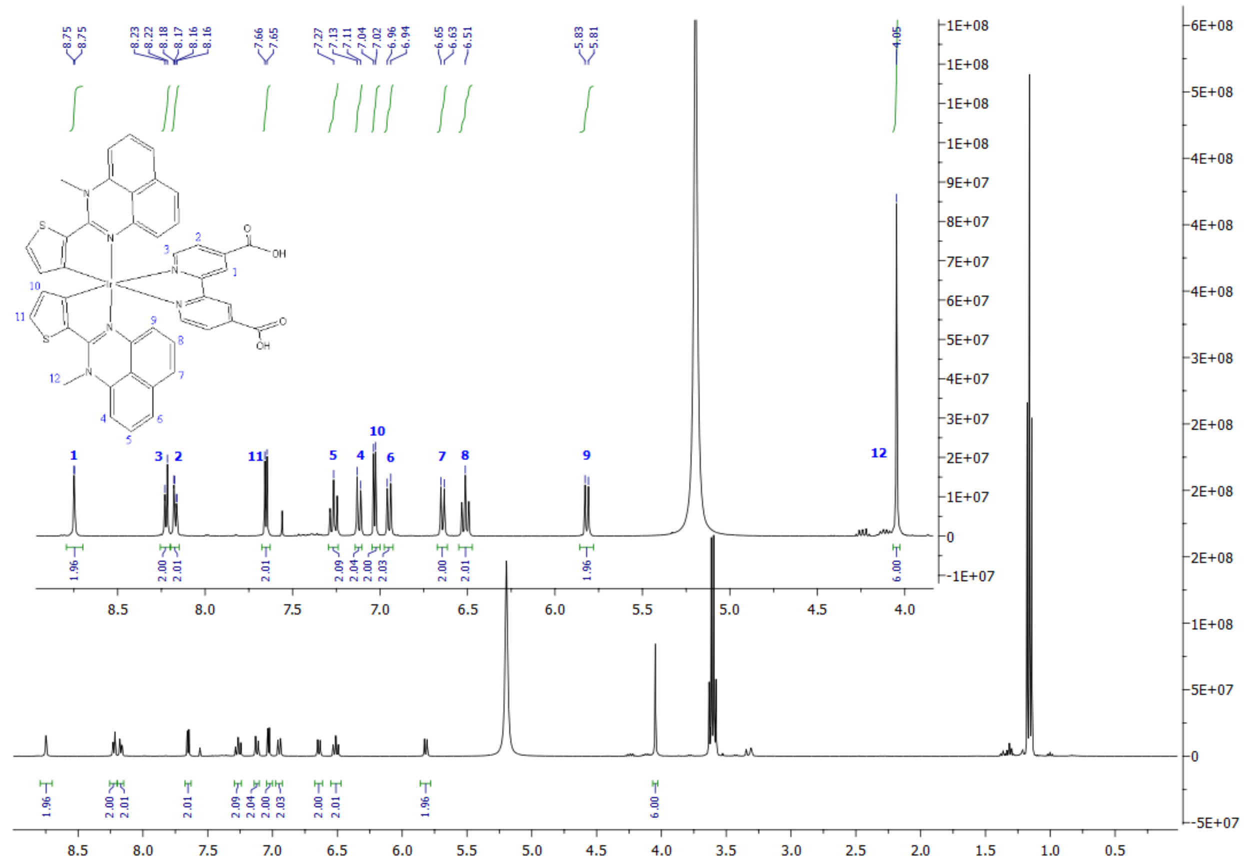

Figure S7. ^{1}H NMR spectrum of **2** (CD_3OD, 400 MHz, 25°C).

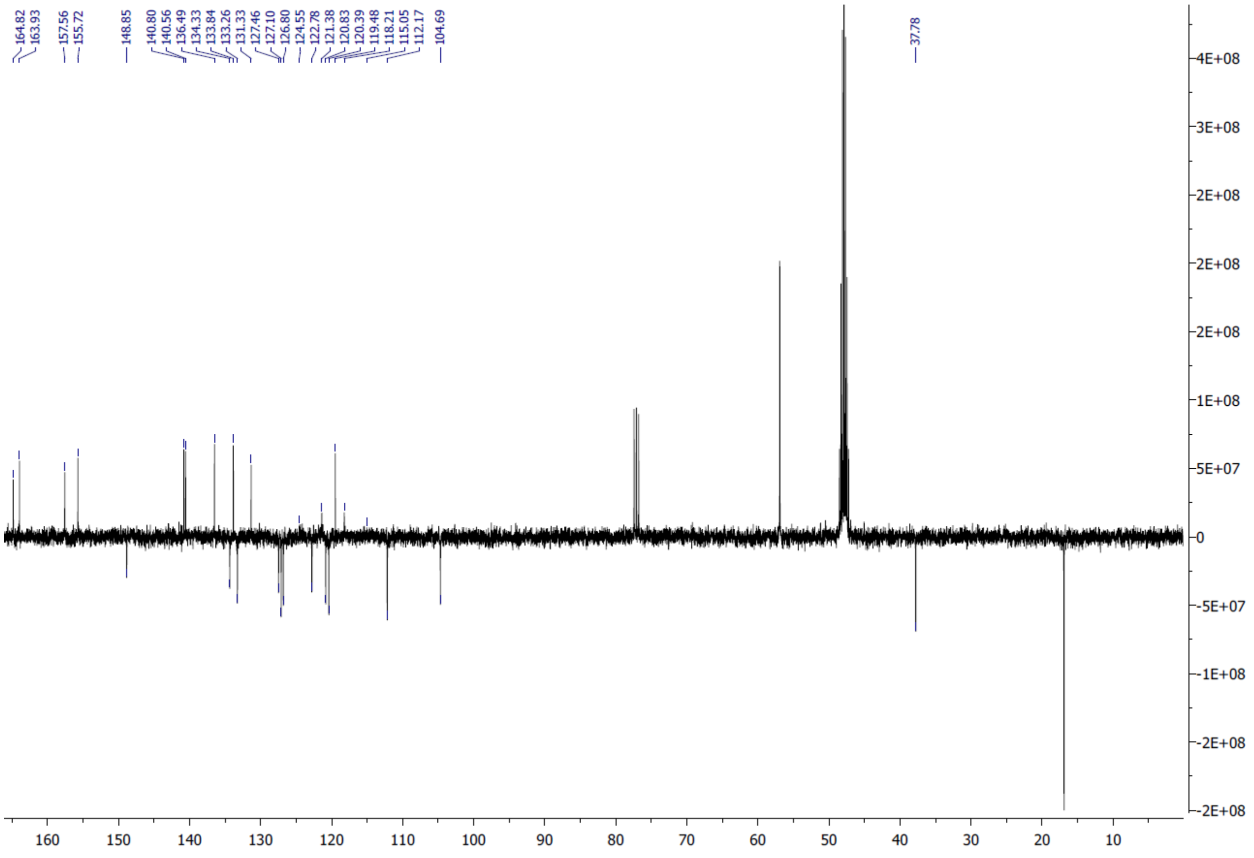

Figure S8. $^{13}C\{^{1}H\}$ NMR spectrum of **2** (CD_3OD, 101 MHz, 25°C).

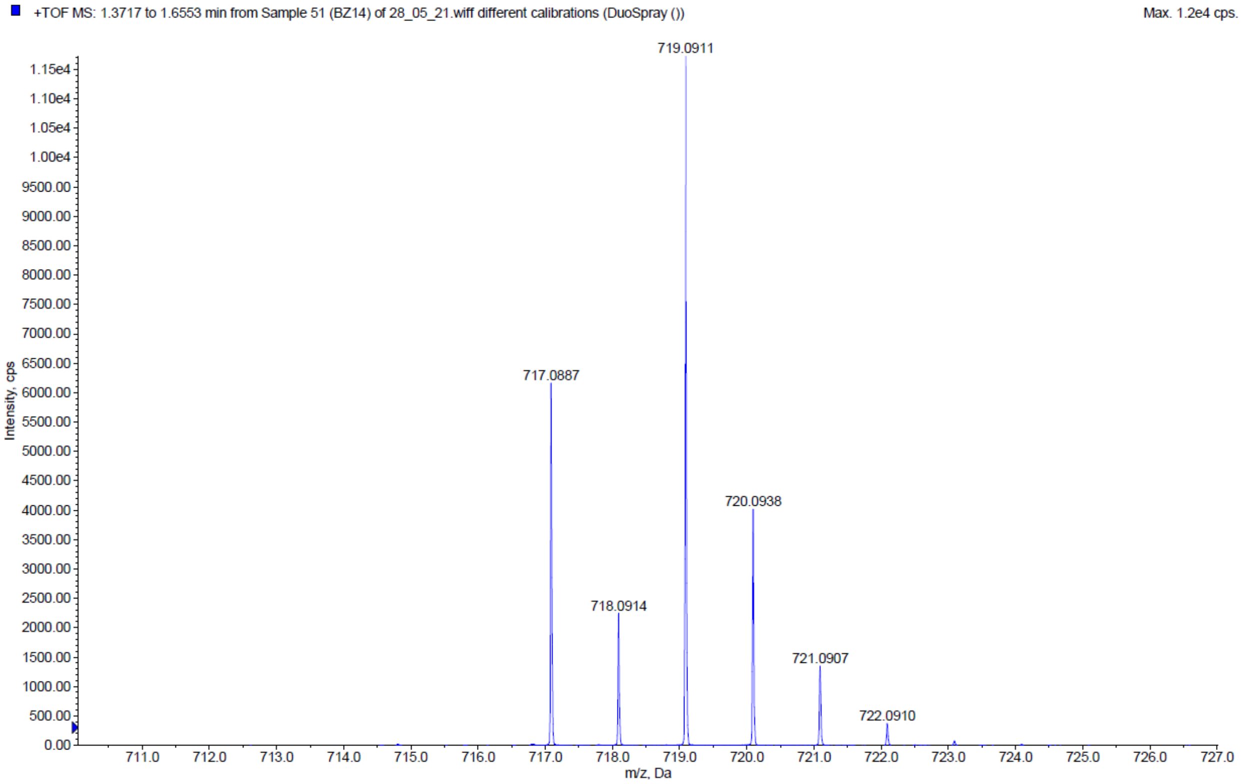

Figure S9. Fragment of high-resolution mass-spectrum of **1**.

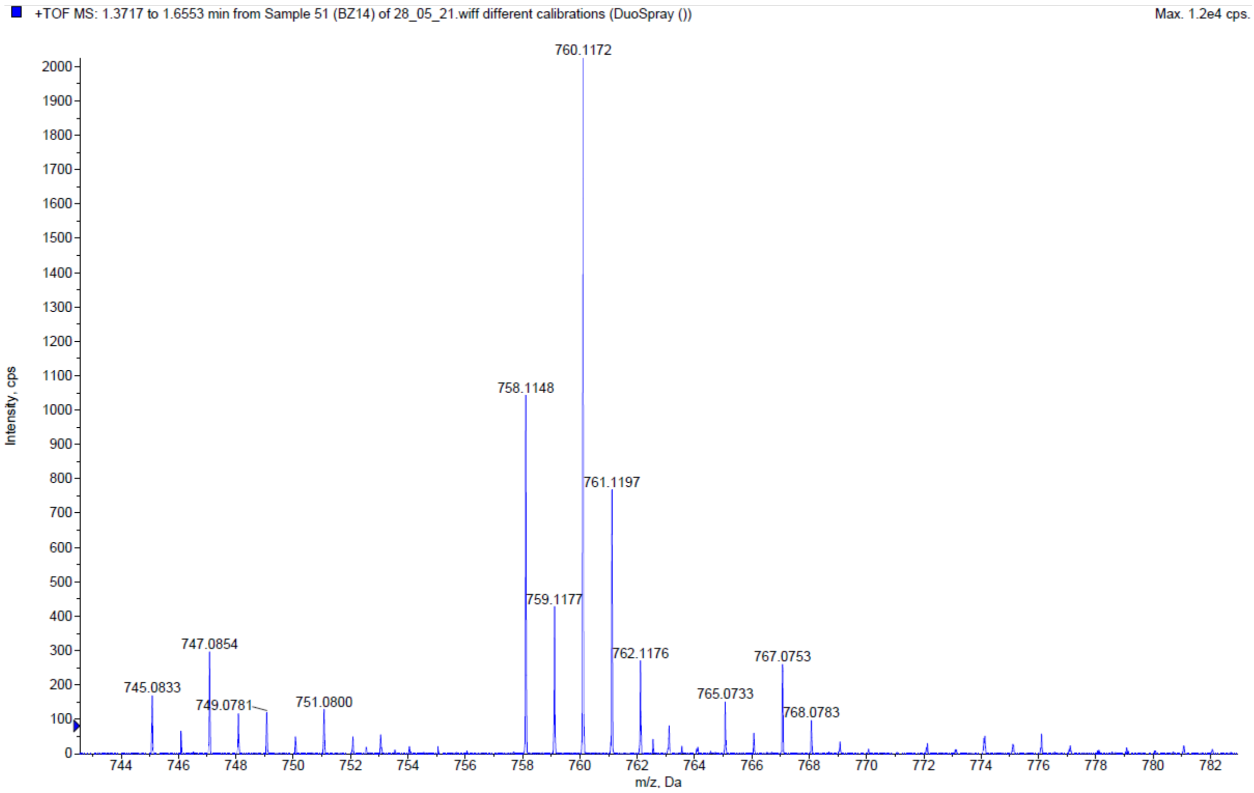

Figure S10. Fragment of high-resolution mass-spectrum of **1**.

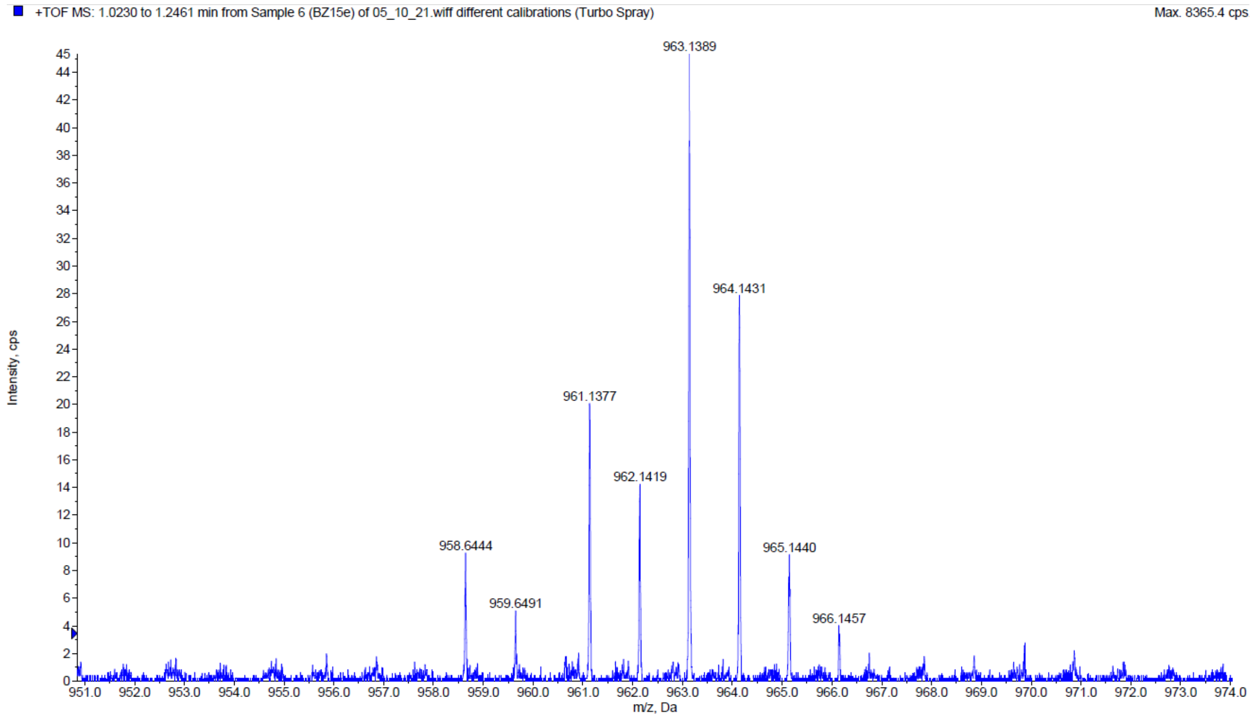

Figure S11. Fragment of high-resolution mass-spectrum of **2**.

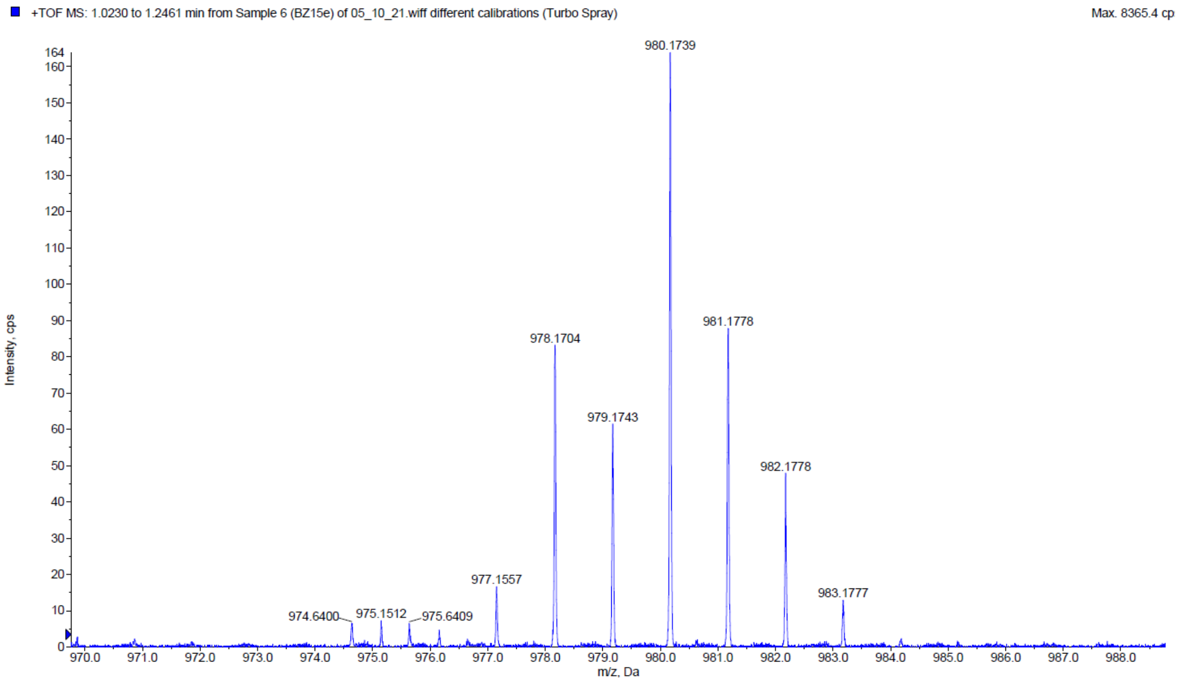

Figure S12. Fragment of high-resolution mass-spectrum of **2**.

2. X-ray crystallography

Table S1. Details of the X-ray crystal data collection and structure refinement for **L1**, **L2**, **1** and **2**.

Compound	**L1**	**L2**	**1**	**2**
Formula	$C_{15}H_{10}N_2S{\cdot}CH_2Cl_2$	$C_{16}H_{12}N_2S$	$C_{64}H_{44}N_8Cl_2S_4Ir_2$	$C_{44}H_{30}N_6O_4S_2Ir^{+}{\cdot}PF_6^{-}{\cdot}$ ${\cdot}3CH_3OH{\cdot}3CH_2Cl_2$
M_w	335.24	3824.08	1508.61	1458.93
Temperature (K)	100(2)	100(2)	100(2)	100(2)
Size (mm)	0.10 x 0.12 x 0.20	0.16 x 0.20 x 0.25	0.01 x 0.08 x 0.12	0.15 x 0.17 x 0.24
Cryst. system	monoclinic	triclinic	monoclinic	triclinic
Space group	$P2_1/c$	P-1	$P2_1/c$	P-1
a (Å)	10.9054(5)	7.7813(4)	31.2214(18)	11.3183(5)
b (Å)	14.4126(6)	11.5947(5)	12.4801(7)	13.0884(7)
c (Å)	9.6793(4)	13.6577(6)	16.8310(9)	20.3399(9)
α (°)	90	90	90	106.294(2)
β (°)	103.512(2)	93.067(2)	103.036(2)	103.709(1)
γ (°)	90	90	90	90.068(2)
V (Å^3)	1479.24(11)	1230.46(10)	6389.1(6)	2802.3(2)
Z	4	4	4	2
θ range (deg)	$2.39 < \theta < 30.00$	$2.31 < \theta < 29.50$	$1.77 < \theta < 30.9$	$1.90 < \theta < 33.75$
collected/unique rflns	18307 / 4305	14031 / 3416	80319 / 20156	138712 / 21801
Completeness to θ (%)	99.7	99.7	99.8	97.2
data/restraints/params	4305 / 0 / 238	3416 / 1 / 191	20156 / 0 / 725	21801 / 0 / 725
Goodness of fit on F^2	1.061	1.036	1.064	1.041
Final *R* indices	R1 = 0.0345	R1 = 0.0394	R1 = 0.0501	R1 = 0.0300
($I > 2\sigma(I)$)	wR2 = 0.0832	wR2 = 0.0957	wR2 = 0.1065	wR2 = 0.0737
Largest diff peak/hole (e/Å^3)	0.47 / −0.29	0.30 / −0.20	5.69 / −4.68	2.30 / −1.88

Table S2. Selected bond lengths (Å) and angles (°) in structures of complexes **1** and **2** and in calculated structure of complex **1.**

**1exp**	$Ir_1–C_1$	$Ir_1–C_2$	$Ir_1–N_1$	$Ir_1–N_2$	$Ir_1–Cl_1$	$Ir_1–Cl_2$
	1.961(6)	1.964(6)	2.088(5)	2.098(5)	2.5109(14)	2.5199(14)
	$Cl_1–Ir_1–Cl_2$	$C_1–Ir_1–C_2$	$C_1–Ir_1–N_2$	$C_2–Ir_1–N_1$	$C_1–Ir_1–Cl_1$	$C_2–Ir_1–Cl_2$
	79.99(4)	93.0(2)	95.3(2)	94.4(2)	93.77(16)	93.28(17)
**1calc**	$Ir_1–C_1$	$Ir_1–C_2$	$Ir_1–N_1$	$Ir_1–N_2$	$Ir_1–Cl_1$	$Ir_1–Cl_2$
	1.976	1.976	2.131	2.134	2.631	2.635
	$Cl_1–Ir_1–Cl_2$	$C_1–Ir_1–C_2$	$C_1–Ir_1–N_2$	$C_2–Ir_1–N_1$	$C_1–Ir_1–Cl_1$	$C_2–Ir_1–Cl_2$
	78.8	96.5	95.9	96.3	92.7	92.0
**2**	$Ir_1–C_1$	$Ir_1–C_{20}$	$Ir_1–N_1$	$Ir_1–N_2$	$Ir_1–N_3$	$Ir_1–N_4$
	1.986(2)	1.977(2)	2.0921(18)	2.0928(18)	2.1628(17)	2.1404(17)
	$N_1–Ir_1–C_2$	$C_1–Ir_1–C_2$	$C_1–Ir_1–N_2$	$C_1–Ir_1–N_3$	$C_2–Ir_1–N_4$	$C_2–Ir_1–N_3$
	93.08(8)	87.37(8)	93.60(8)	100.25(7)	97.42(7)	169.86(8)

Table S3. Dihedral angles between the aryl and perimidine units (φ) and angles between the N–CH_3 bond and the perimidine plane (θ) in the structures of **L1** and **L2**.

	**L1**	**L2**
φ, °	12.13(4)	67.42(13)
θ, °	-	5.08(7)

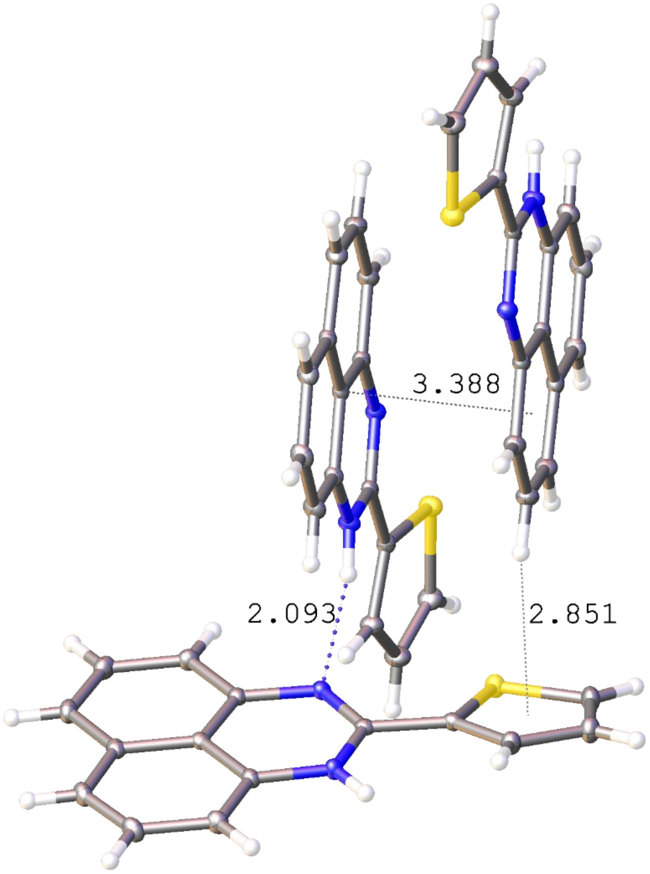

Figure S13. Hydrogen bonding, π···π and C–H···π interactions in the crystal of **L1**.

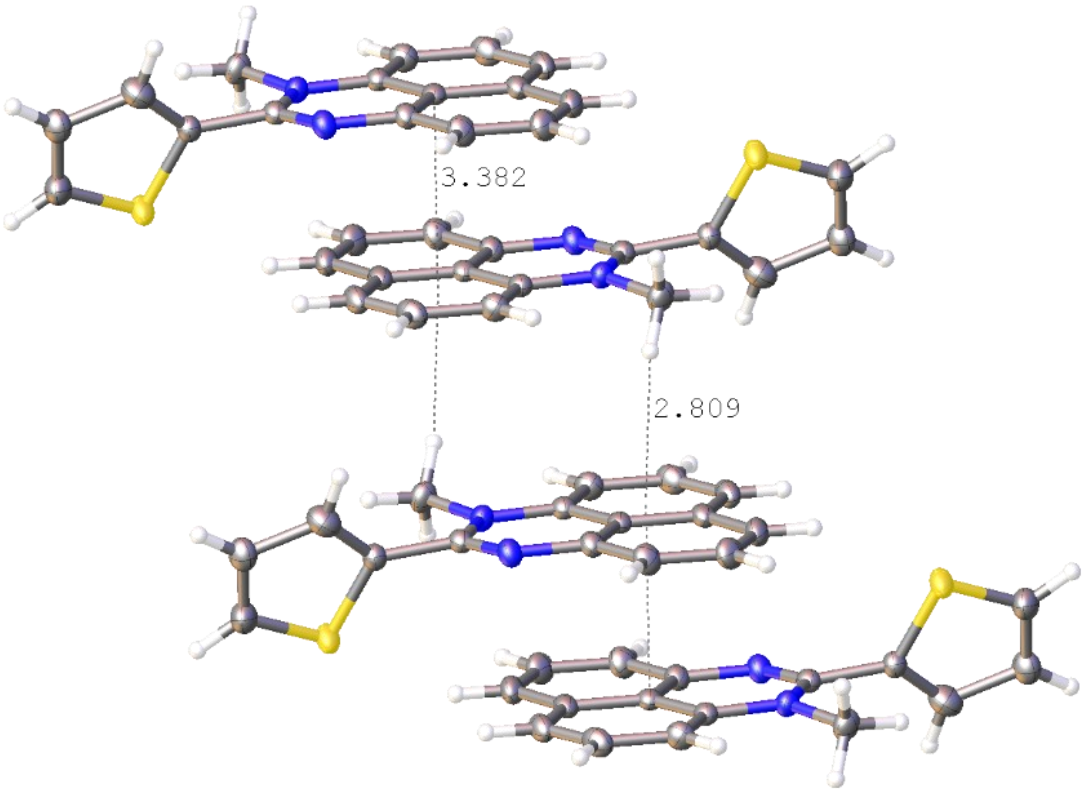

Figure S14. π···π and C–H···π interactions in the crystal of **L2**.

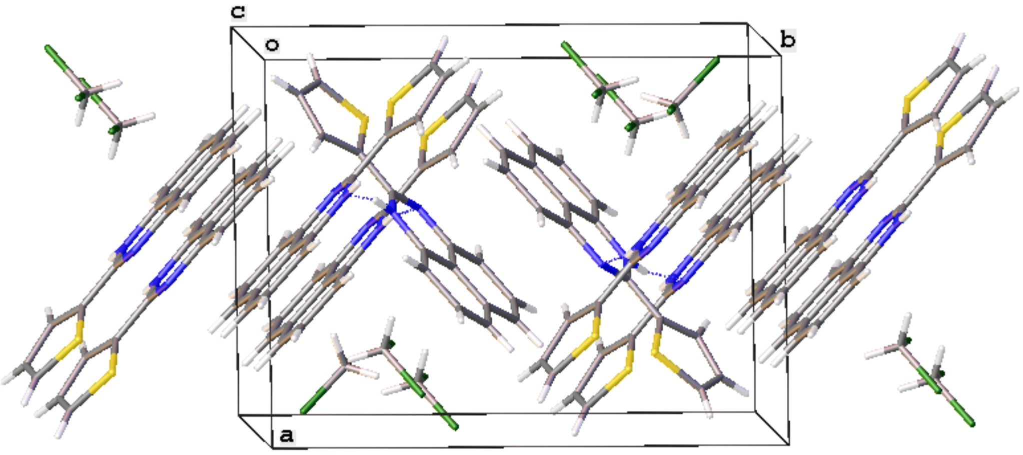

Figure S15. The fragment of the crystal packing of **L1**.

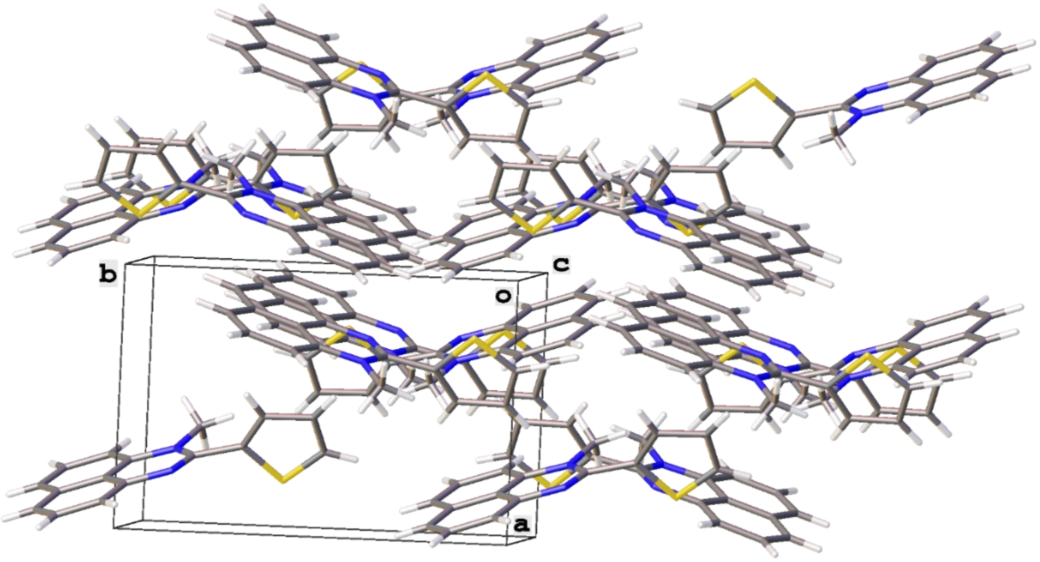

Figure S16. The fragment of the crystal packing of **L2**.

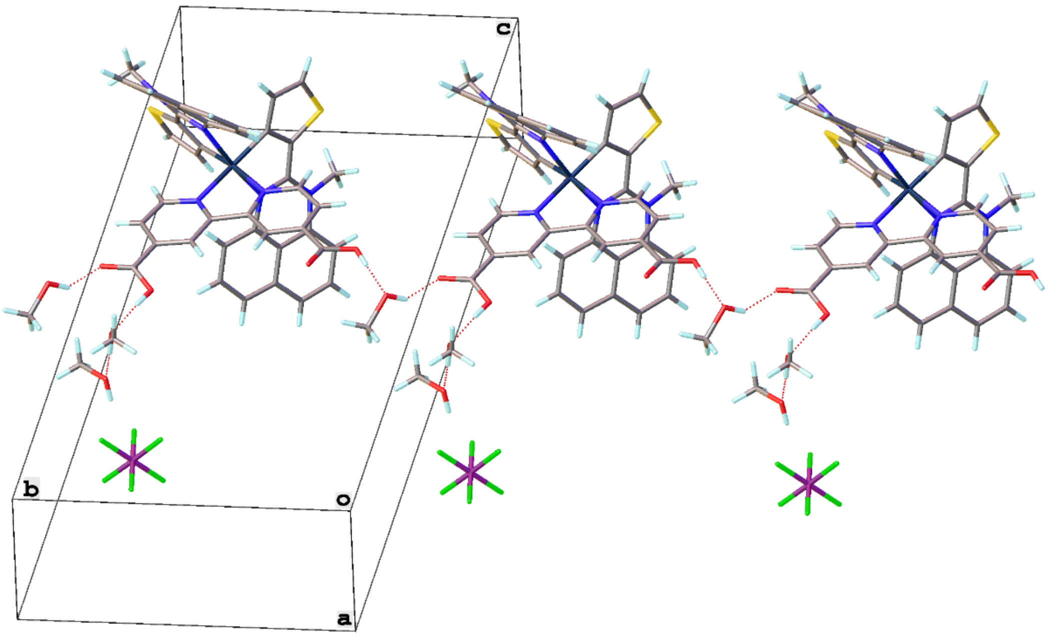

Figure S17. The fragment of the crystal packing of **2**.

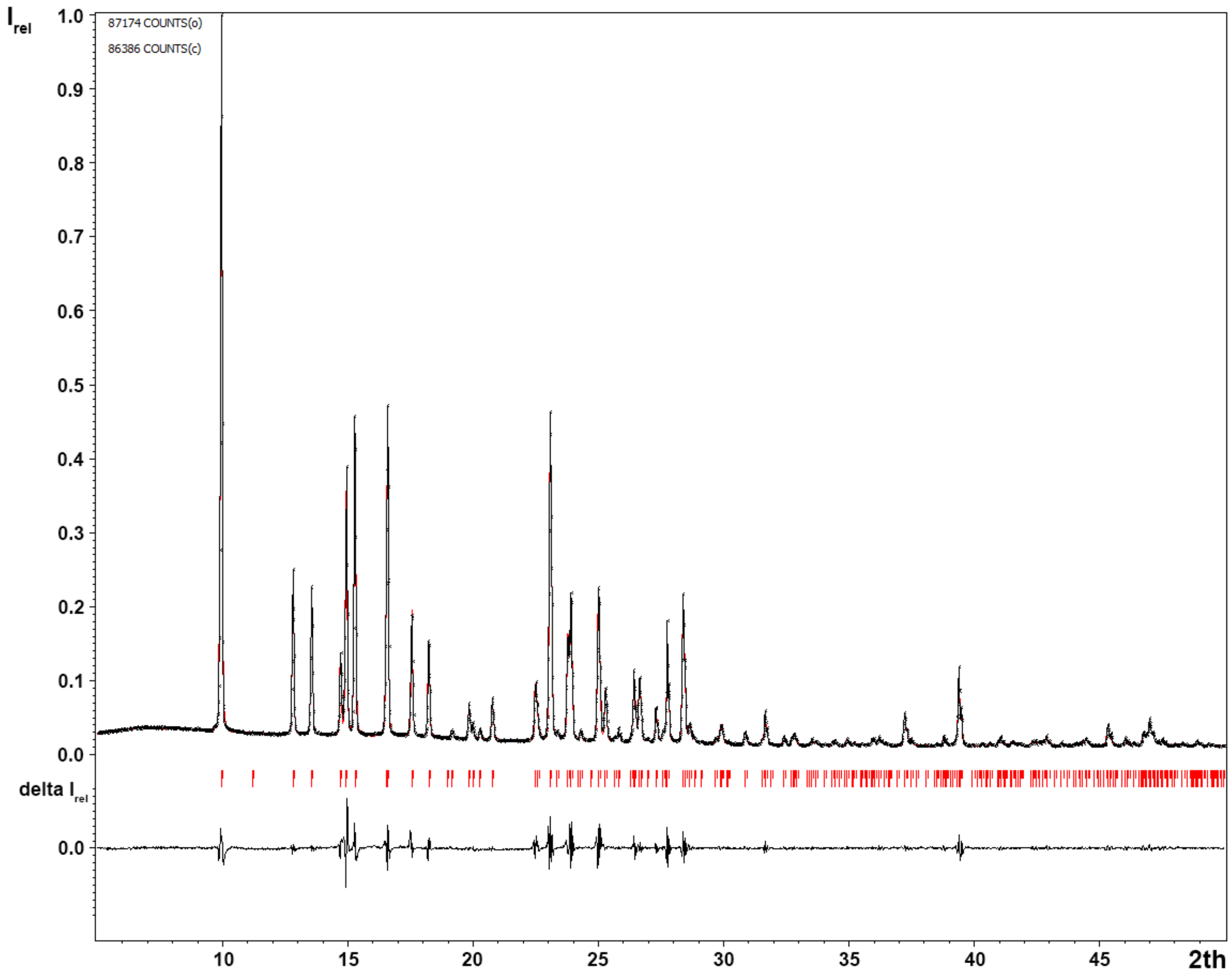

Figure S18. Experimental (black) and simulated (red) X-ray powder diffraction patterns of **L2**.

Table S4. Unit cell parameters for **L2** based on single-crystal and powder X-ray data and details of their refinement.

	a, Å	*b*, Å	*c*, Å	β, °	V, Å^3	FOM	2θ-window	peaks
powder	7.894(1)	11.566(2)	13.794(3)	93.43(1)	1257.3(3)	100.00	0.06	77/78
single-crystal	7.7813(4)	11.5947(5)	13.6577(6)	93.067(2)	1230.46(10)			

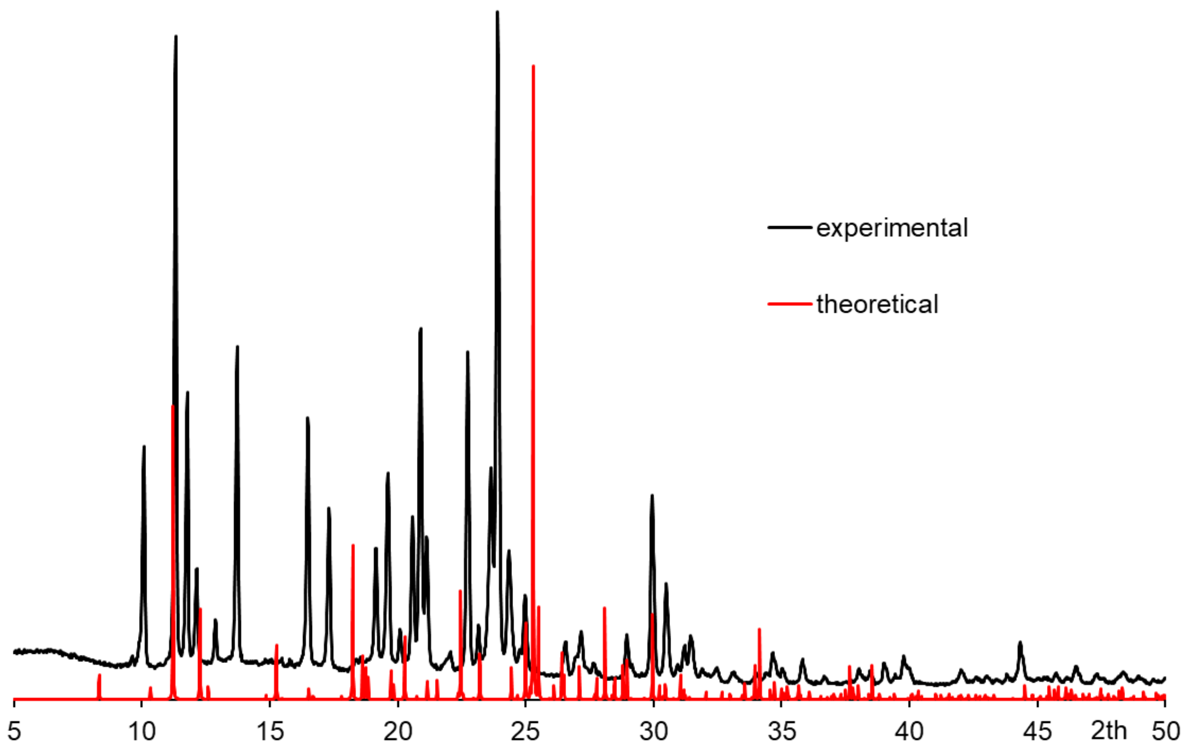

Figure S19. Experimental (black) and simulated (red) X-ray powder diffraction patterns of **L1**.

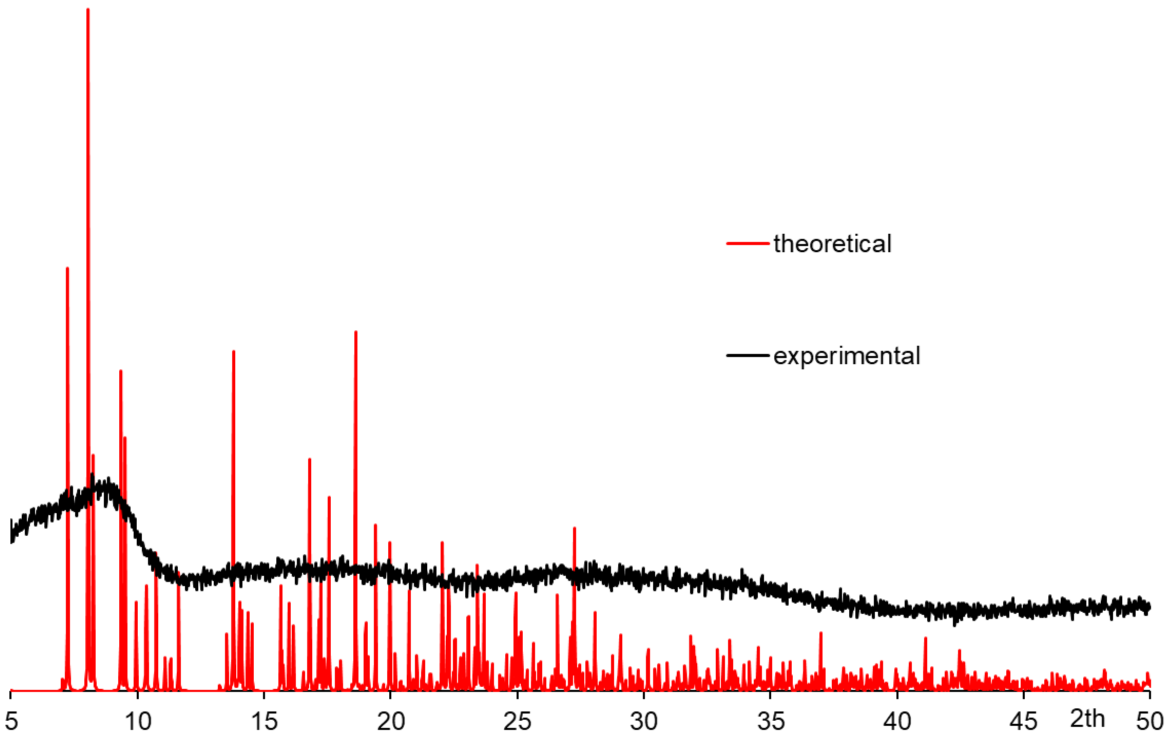

Figure S20. Experimental (black) and simulated (red) X-ray powder diffraction patterns of **2**.

3. Electrochemical and photovoltaic data

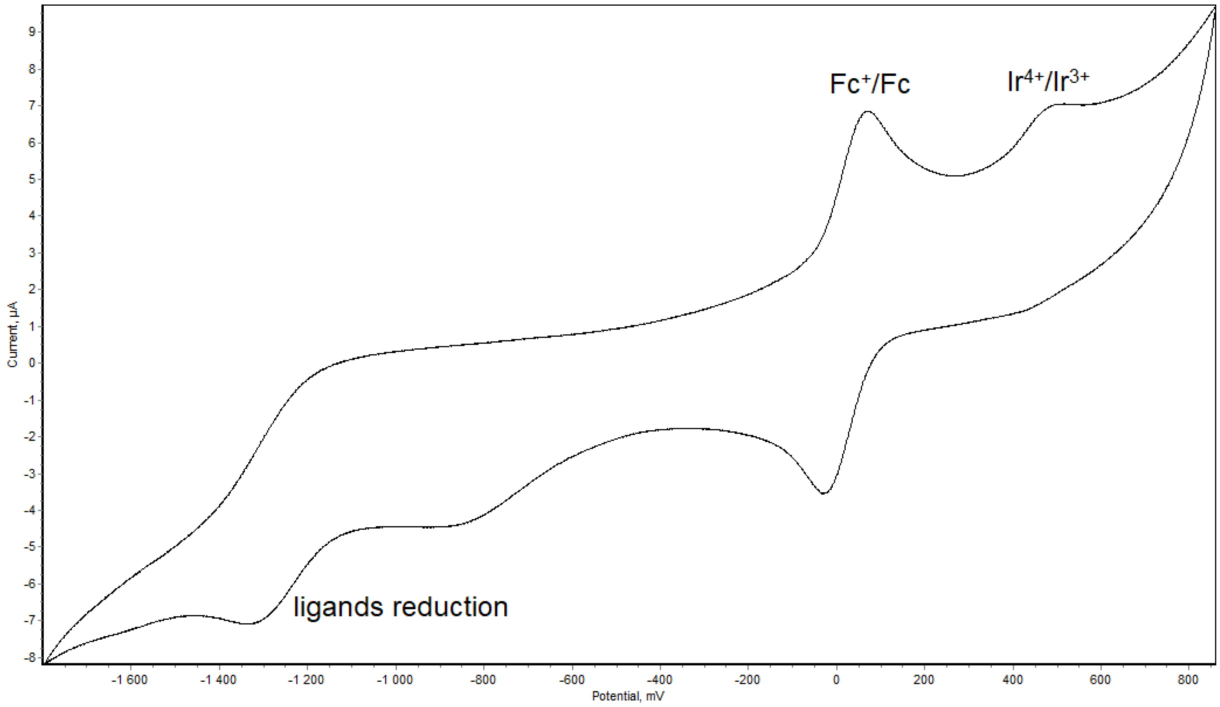

Figure S21. Cyclic voltammogram of **2** recorded in Ar-saturated acetonitrile at 20°C *versus* ferrocene at a scan rate of 100 mV/s (electrolyte 0.1 M NBu_4ClO_4).

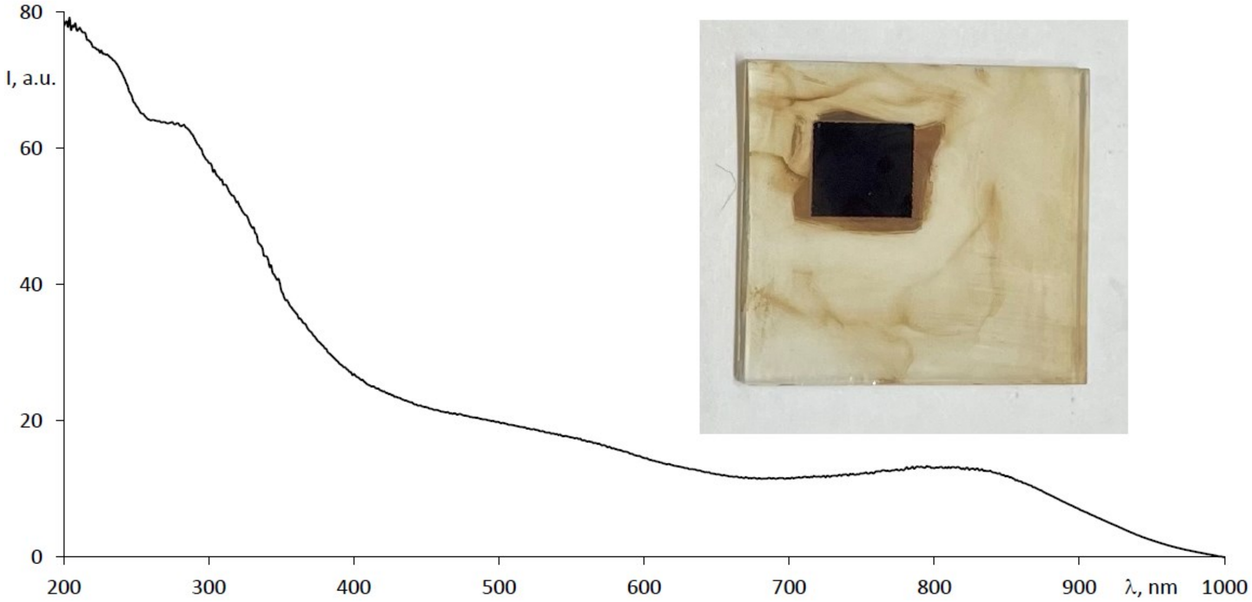

Figure S22. Diffuse reflectance spectrum of a TiO_2 photoanode sensitized by complex **2** measured at the TiO_2 side of the electrode.

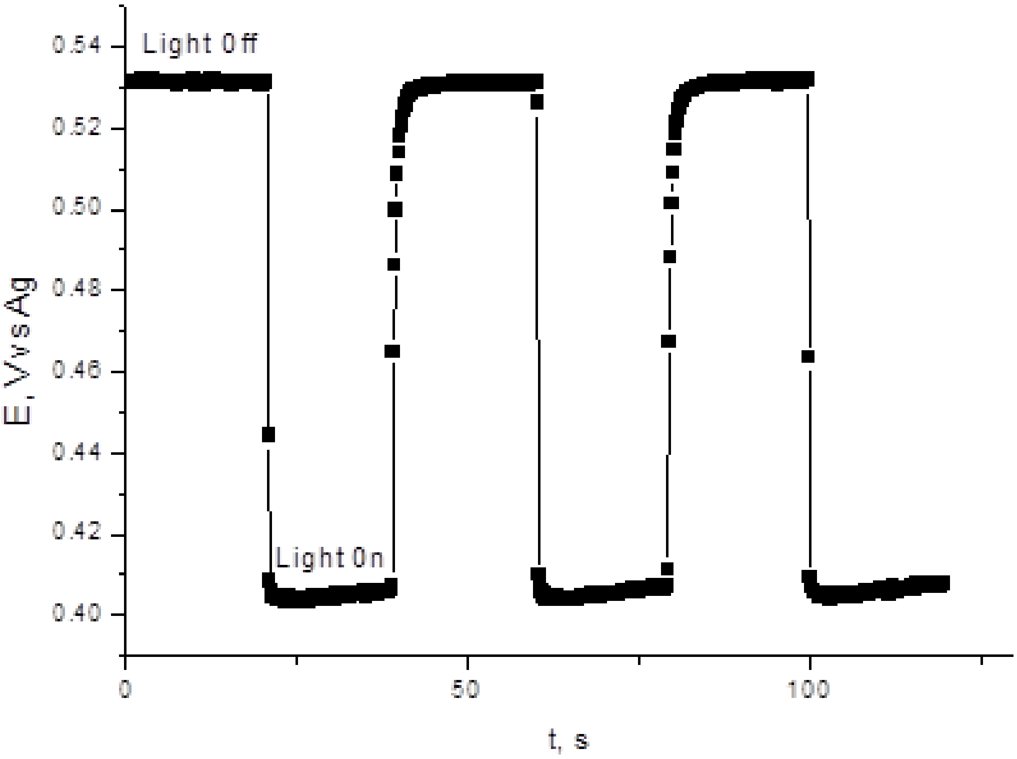

Figure S23. Time dependence of potential of TiO_2 photoanode sensitized by complex **2** in the dark and on exposure to AM 1.5 G simulated solar light (100 mW cm^{-2}) in acetonitrile solution in the presence of 0.5 M LiI + 0.05 M I_2.

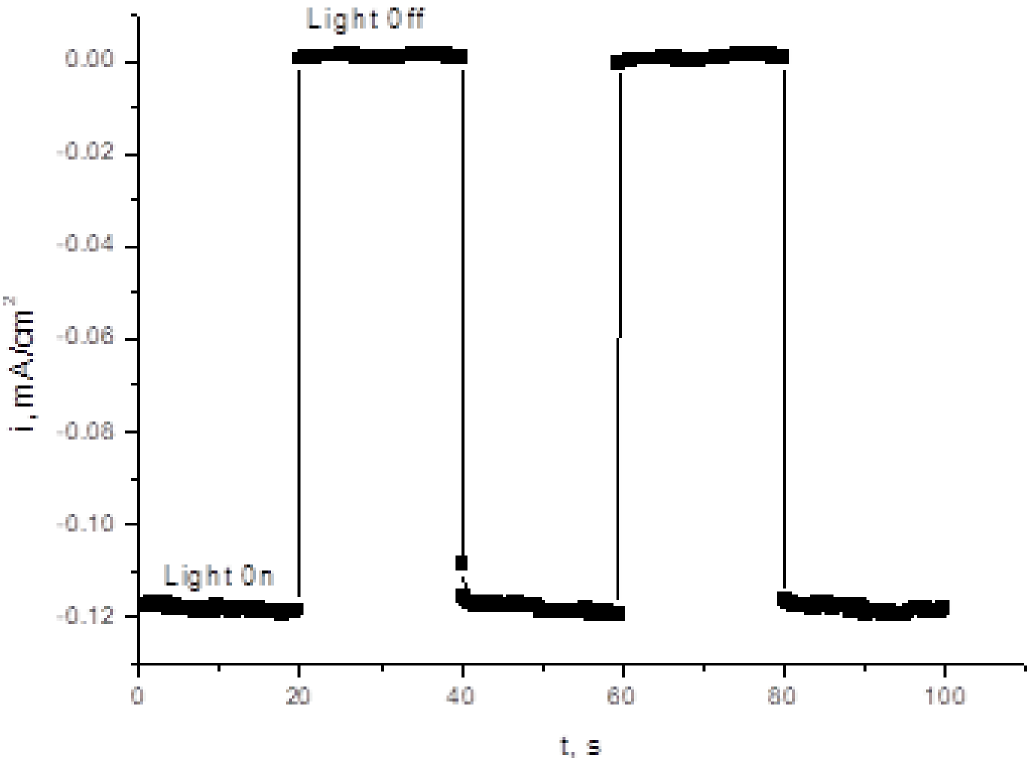

Figure S24. Time dependence of current at short circuit for PECC with TiO_2 photoanode sensitized by complex **2** in the dark and on exposure to AM 1.5 G simulated solar light (100 mW cm^{-2}) in acetonitrile solution in the presence of 0.5 M LiI + 0.05 M I_2.

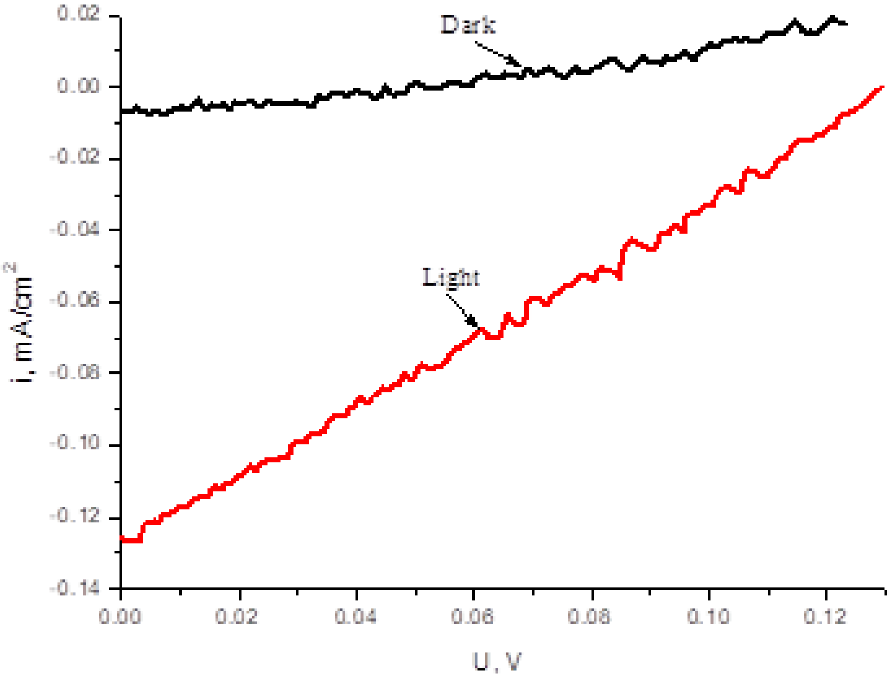

Figure S25. Current–voltage characteristic of a TiO_2 photoanode sensitized by complex **2** under AM 1.5 G simulated solar light (100 mW cm^{-2}) (*red*) and in the dark (*black*) in acetonitrile solution of 0.5 M LiI + 0.05 M I_2.

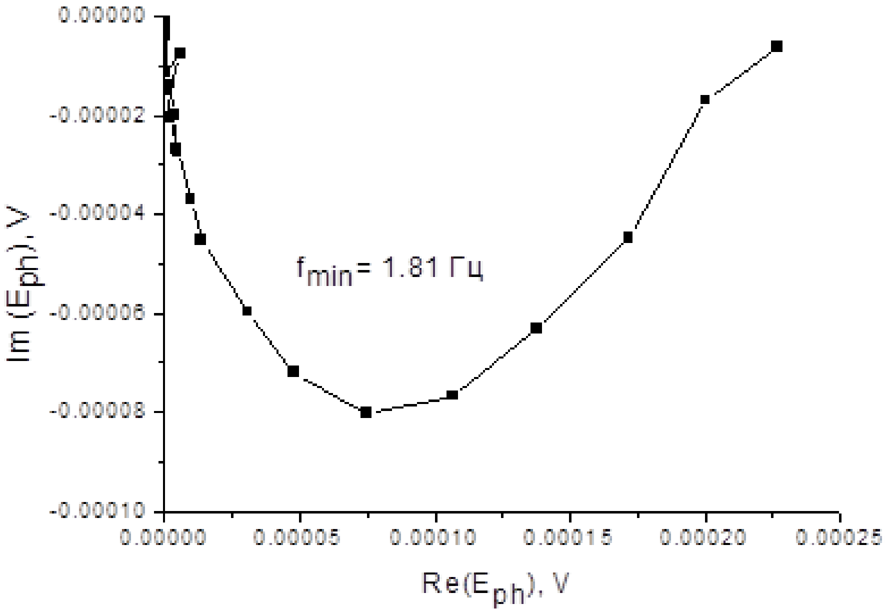

Figure S26. Intensity-modulated photovoltage spectrum (IMVS) for TiO_2 photoanode sensitized by complex **2**. Illumination by monochromatic light (428 nm) at the power of 12 mW.

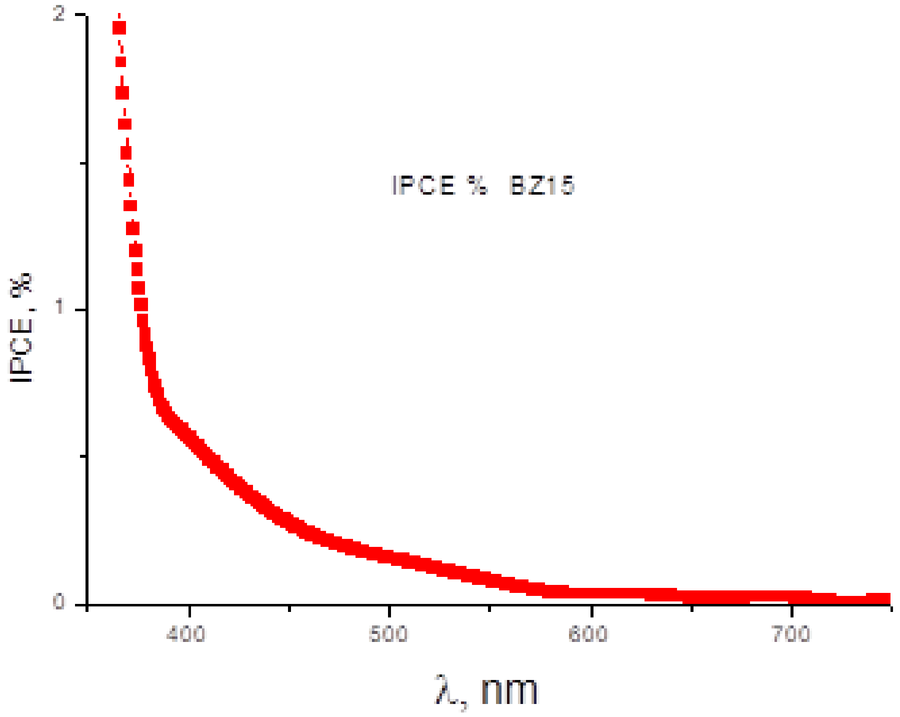

Figure S27. Incident photon-to-electron conversion efficiency (IPCE) spectrum of TiO_2 photoanode sensitized by complex **2** under AM 1.5 G simulated solar light (100 mW cm^{-2}).

4. Computational results

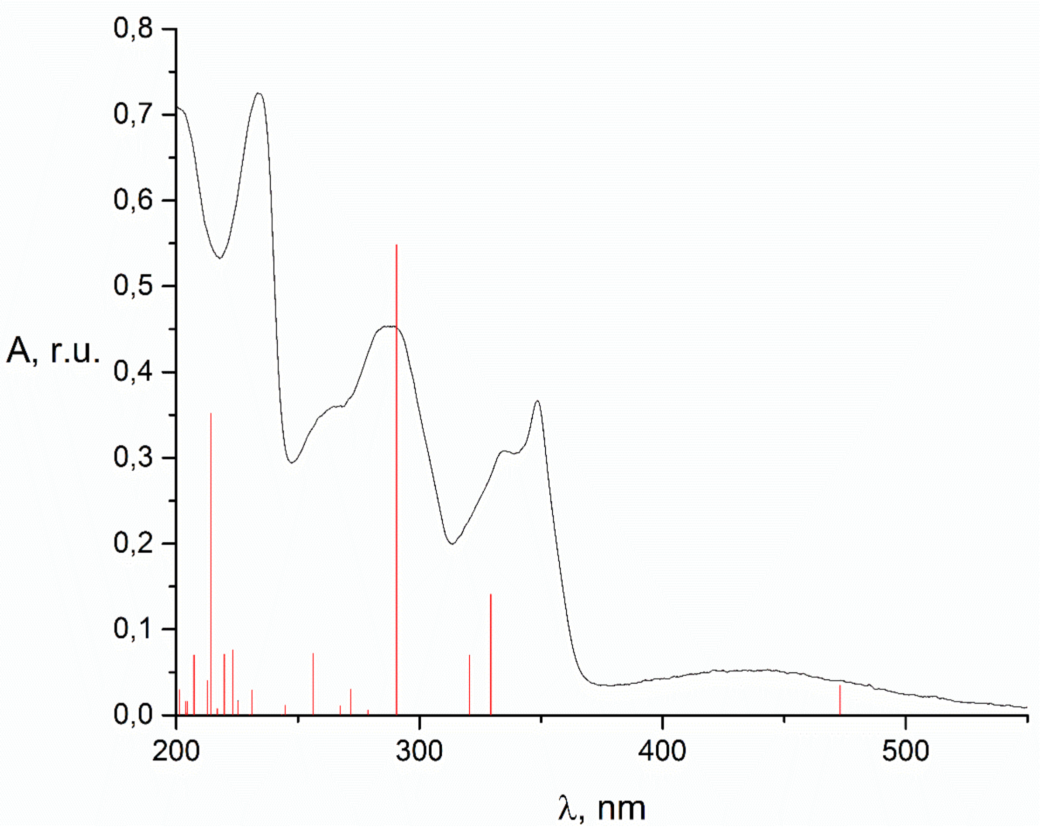

Figure S28. TDDFT electronic (*red*) and experimental (*black*) spectra of **L1**.

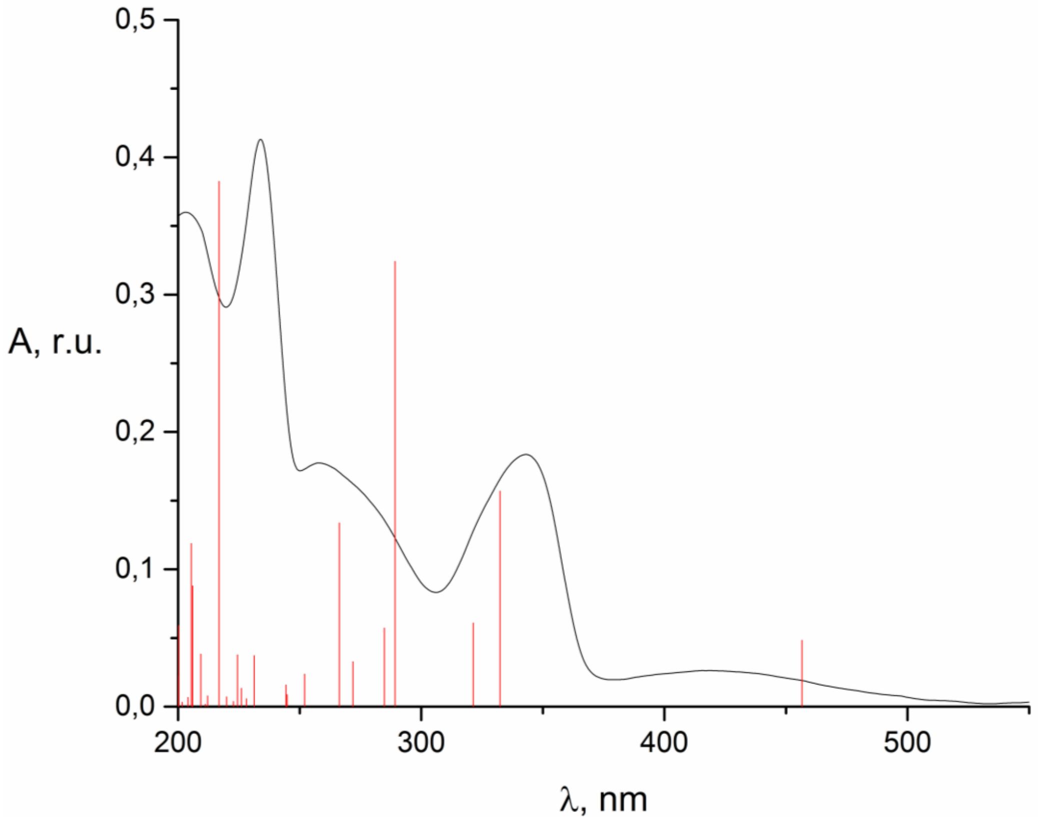

Figure S29. TDDFT electronic (*red*) and experimental (*black*) spectra of **L2**.

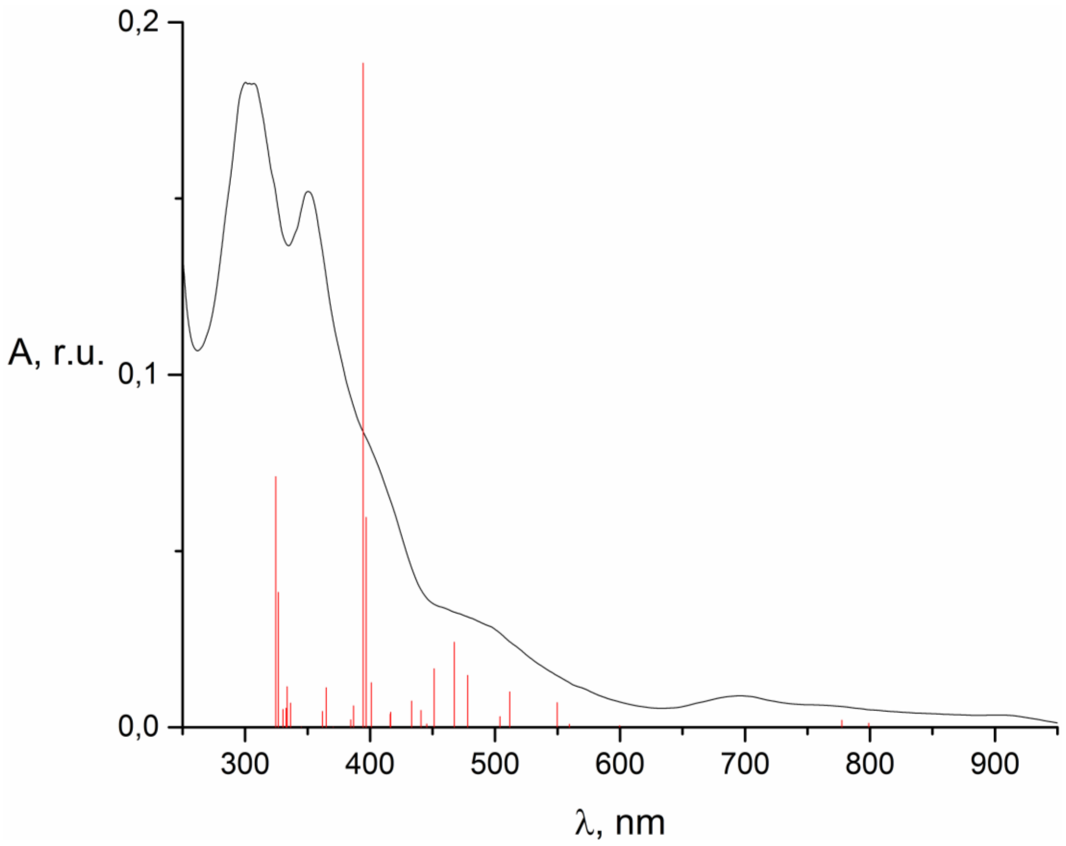

Figure S30. TDDFT electronic (*red*) and experimental (*black*) spectra of **2**.

Table S5. TDDFT singlet excited states (λ > 500 nm) for **2**.

Complex	State	λ / nm (*f*)	Dominant monoexcitations
	S1	799 (0.0011)	H → L (88%) {*π(C^N) → π*(N^N)*}
	S2	777 (0.0020)	H-1 → L (88%) {*π(C^N) → π*(N^N)*}
	S3	600 (0.0004)	H-2 → L (98%) {*π(C^N) → π*(N^N), d(Ir) → π*(N^N)*}
**1**	S4	560 (0.0008)	H → L+1 (91%) {*π(C^N) → π*(N^N)*}
	S5	550 (0.0070)	H-1 → L+1 (91%) {*π(C^N) → π*(N^N)*}
	S6	511 (0.0100)	H → L+2 (92%) {*π(C^N) → π*(N^N)*}
	S7	504 (0.0030)	H-1 → L+2 (91%) {*π(C^N) → π*(N^N)*}
